# Generative AI-augmented transcriptomic and microbiome analysis across inflammatory and fibrotic disease states in Crohn’s disease

**DOI:** 10.3389/frai.2026.1881820

**Published:** 2026-08-04

**Authors:** Daryll Philip, Daniela Santos, Sudip Mondal, Haneen Alomar, Georgios Gkoutos, Animesh Acharjee

**Affiliations:** 1 Cancer and Genomic Sciences, School of Medical Sciences, College of Medicine and Health, University of Birmingham Dubai, Dubai, United Arab Emirates; 2 Cancer and Genomic Sciences, School of Medical Sciences, College of Medicine and Health, University of Birmingham, Birmingham, United Kingdom; 3 Centre for Health Data Research, University of Birmingham, Birmingham, United Kingdom; 4 Institute of Translational Medicine, University Hospitals Birmingham NHS, Foundation Trust, Birmingham, United Kingdom; 5 Department of Research Laboratories, King Faisal Specialist Hospital & Research Centre, Jeddah, Saudi Arabia

**Keywords:** Crohn’s disease, fibrosis, generative AI, inflammatory bowel disease, large language modelling, machine learning, microbiome, transcriptomics

## Abstract

**Introduction:**

Intestinal fibrosis is a major complication of Crohn’s disease (CD), a subtype of inflammatory bowel disease (IBD) driven by chronic inflammation and resulting in irreversible structural damage requiring surgery. However, the molecular differences between inflammatory and fibrotic CD remain poorly defined.

**Methods:**

Here, we developed an integrated multi-omics framework combining transcriptomics, microbiome analysis, and generative AI to characterise transcriptomic differences across non-IBD (*n* = 176), baseline CD (*n* = 187), and fibrosis CD (*n* = 85) tissues. Bulk and single-cell RNA-seq and 16S rRNA datasets were integrated, and machine learning identified disease-stage associated features.

**Results:**

A shared set of 43 genes between baseline and fibrotic CD was organised into three modules: Module 1 (S100A8, TREM1, CXCL1) linked to innate immune activation which was upregulated in fibrosis CD; Module 2 (FABP6, MGAM, ALDOB) reflecting epithelial metabolic dysfunction which was upregulated in baseline CD; and Module 3 (CHI3L1, SAA2-SAA4, IL1RN) associated with epithelial stress and loss of barrier integrity. GSVA highlighted LCN2 and MMP3 across disease states. Microbiome analysis showed depletion of SCFA-producing genera (*Faecalibacterium*, *Anaerostipes*, *Coprococcus*, *Ruminococcus*) and enrichment of *Bilophila* and *Bacteroides*. Notably, LLM-guided augmentation improved model stability and facilitated the identification of key fibrosis-associated genes, including IL-23R, TNF-α, and TGF-β.

**Discussion:**

These findings suggest that intestinal fibrosis in CD does not represent a separate molecular state, but a reconfigured inflammatory condition characterised by persistent immune activation, epithelial dysfunction, and altered host-microbiome interactions.

## 1 Introduction

Inflammatory bowel disease (IBD) is a chronic inflammatory condition of the gastrointestinal tract that encompasses Ulcerative Colitis (UC) and Crohn’s Disease (CD). The global burden of IBD has risen markedly over the past decades, with the Global Burden of Disease (GBD) 2017 study estimating more than 6.8 million prevalent cases worldwide and continuing increases in incidence globally (Wang et al., 2023; Zhang et al., 2024a). CD is characterised by transmural inflammation and can affect any part of the gastrointestinal tract, but primarily involves the ileum and colon. Its global prevalence is estimated at approximately 4 million cases, with an incidence rate of up to 84 per 100,000 individuals in industrialised regions (Heydari et al., 2025).

Patients with IBD frequently experience impaired quality of life due to chronic symptoms and complications. One of the most serious complications of CD is intestinal fibrosis, a process marked by fibroblast proliferation and excessive extracellular matrix (ECM) deposition that follows recurrent cycles of inflammation and repair. Fibrosis leads to structural remodelling of the bowel wall, resulting in strictures and bowel obstruction that often require surgical intervention (Rieder et al., 2024; Dudek and Talar-Wojnarowska, 2024).

Existing treatments for IBD, such as aminosalicylates, corticosteroids, immunomodulators, and biologic agents such as anti-TNF and anti-integrin therapies, primarily target inflammation. However, they have a limited direct impact on the fibrogenic mechanisms associated with stricture formation (Rieder et al., 2016; Amamou et al., 2025; Pieczarkowski et al., 2020). Consequently, intestinal fibrosis represents a major unmet need in IBD care. Experimental antifibrotic approaches, primarily evaluated in DSS- or TNBS-induced colitis models, have shown promise. For instance, statins (e.g., simvastatin, pravastatin) attenuate TGF-*β* signalling and collagen synthesis while promoting fibroblast apoptosis (Abe et al., 2012). Additionally, pirfenidone inhibits the TGF-β/SMAD and mTOR/p70S6K pathways, thereby suppressing fibroblast activation and ECM accumulation (Sun et al., 2018). Despite promising preclinical results, no antifibrotic therapy has been approved, emphasising the need to effectively address the underlying fibrotic pathways associated with fibrotic complications.

Once fibrosis develops, it is generally considered difficult to reverse. Notably, up to 70% of patients with transmural inflammation develop stricturing complications after 10 years of diagnosis. At present, surgical intervention remains a primary treatment option for established intestinal fibrosis, although it does not prevent recurrence (Lin et al., 2022; Biel et al., 2023).

Transcriptomic studies suggest that intestinal fibrosis in CD reflects interactions between mesenchymal and immune cells, involving both persistent inflammatory signalling and additional stromal and tissue-remodelling processes. Fibroblasts, particularly when activated into myofibroblasts, play a central role by driving ECM deposition, with additional contributions from telocytes, fibrocytes, epithelial and endothelial cells undergoing Epithelial to Mesenchymal Transition (EMT/EndMT) (Wu et al., 2023; Mukherjee et al., 2023; Giannandrea and Parks, 2014). Immune cells also drive fibrosis by activating stromal cells such as macrophages, T cells, monocytes, and innate lymphoid cells, which secrete cytokines, such as TGF-*β*, IL-13, and IL-17, that promote fibroblast activation (Zhang et al., 2025).

In contrast, non-fibrotic CD is predominantly characterised by inflammatory responses, with Th1 and Th17 cells producing TNF-*α*, IFN-*γ*, and IL-17, leading to epithelial injury and neutrophil recruitment. Macrophages exhibit a proinflammatory M1-like phenotype, while regulatory T cells (Tregs) are impaired and fail to control inflammation (Huang et al., 2023).

Beyond transcriptomic analyses, complementary high-throughput approaches have further characterised the biology underlying CD and its fibrotic complications. Large-scale multi-omics profiling from the Integrative Human Microbiome Project (IBDMDB) demonstrated that CD is associated with coordinated alterations in microbial composition, microbial functional capacity, and host-derived metabolites, including disruptions in bile acid metabolism and short-chain fatty acid pathways, highlighting the metabolic dimension of intestinal inflammation (Lloyd-Price et al., 2019; Proctor et al., 2019). Increasing evidence also implicates the gut microbiota as a contributor to fibrogenesis. Early experimental work demonstrated that transplantation of gut microbiota from healthy rats into germ-free animals could induce collagen deposition at intestinal sites, providing experimental evidence that microbial communities can influence fibrotic responses (Mourelle et al., 1998). In humans, circulating antibodies against microbial antigens have been associated with fibrotic disease phenotypes in CD, suggesting that microbe-host immune interactions may contribute to tissue remodelling (Zhan et al., 2021; Mow et al., 2004). Microbiome profiling studies further support this link, with altered microbial communities observed in fibrotic intestinal regions, including reduced abundance of taxa such as *Lactobacillus*, *Oscillospira*, *Subdoligranulum*, *Hydrogenophaga*, and *Clostridium* (Zhan et al., 2023). Together, these findings indicate that intestinal fibrosis likely reflects the interplay of microbial, metabolic, immune, and stromal processes. However, most studies have examined these biological layers in isolation, limiting our understanding of how these systems contribute to differences observed between inflammatory and fibrotic disease states. An integrative multimodal framework is therefore needed to better characterise the molecular architecture associated with fibrotic disease stages in CD.

Importantly, another key limitation is the cross-sectional nature of available datasets; most transcriptomics and clinical data are collected at a single time point, limiting the ability to document changes in inflammation, tissue remodelling, and treatment responses over time. This is particularly challenging for fibrosis, where early detection is critical, established fibrotic tissue changes are often difficult to reverse, and longitudinal datasets remain limited. To address these challenges, computational approaches can be used to identify molecular patterns consistent with different disease stages using case–control and cross-sectional datasets. In this context, generative models, including large language models (LLMs), provide a framework not only for biologically informed data augmentation but also for reconstructing plausible transcriptomic patterns and recovering low-abundance signals that may be lost during preprocessing (Kwon et al., 2021; Bzdok et al., 2024; Kircher et al., 2022). This approach can improve the stability of downstream modelling and facilitate the identification of disease state-associated molecular differences in sparse datasets.

In this work, we examined the molecular differences among non-IBD, inflammatory CD, and fibrotic CD tissues using an integrated computational analysis of RNA-seq and microbiome datasets. Specifically, we sought to identify transcriptomic signatures associated with disease states and determine the shared and stage-specific gene expression patterns by integrating bulk RNA-seq and single-cell RNA-seq datasets. In parallel, 16S rRNA data were analysed to assess associations between gut microbiome composition and fibrotic disease. In addition, we used an LLM-guided approach to both increase sample size and expand the transcriptomics dataset and optimise the selection of fibrosis-associated genes. Together, this framework aims to assess whether intestinal fibrosis shares molecular characteristics with inflammatory CD or exhibits a distinct molecular profile.

## 2 Methodology

### 2.1 Data acquisition

**Figure 1:**
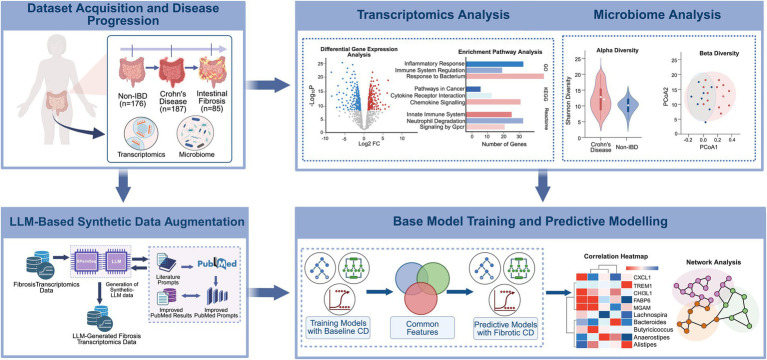
Workflow diagram. Transcriptomics and microbiome data were obtained from colonic tissue samples representing Non-IBD, Crohn’s disease (CD), and fibrosis CD, capturing disease progression from non-fibrotic to fibrotic states. Differential gene expression analysis was performed to identify significant genes, followed by functional pathway enrichment analysis to determine key biological processes and immune-related pathways associated with disease progression. For the microbiome data, alpha diversity (Shannon diversity index) and beta diversity analyses were conducted to assess microbial composition and variation across groups. Baseline CD samples were used to train predictive machine learning models, including eXtreme Gradient Boosting (XGBoost), Random Forest, and Least Absolute Shrinkage and Selection Operator (LASSO). Overlapping features across models were identified to construct robust predictors of fibrotic CD outcomes. In parallel, fibrosis-related data were input into large language models (LLMs), guided by structured prompts informed by sources such as PubMed, to generate LLM-derived fibrosis features. These features were subsequently incorporated into the machine learning models. Finally, correlation-based molecular interaction analysis was performed using heatmaps and network visualisation to identify coordinated gene interactions and key regulatory hubs associated with fibrosis.

An overview of the study workflow is presented in Figure 1. Publicly available transcriptomic and microbiome datasets were retrieved from the Gene Expression Omnibus (GEO), the Single Cell Portal, and the NCBI BioProject. For Crohn’s Disease (CD) bulk RNA-sequencing analyses, datasets GSE230113 (Wang et al., 2024), GSE139179 (Revilla et al., 2021), and GSE83687 (Peters et al., 2017) were included. For fibrosis-associated CD, bulk RNA-seq datasets GSE123141 (Dharmasiri et al., 2021), GSE66207 (Peck et al., 2015), and GSE67250 (Sadler et al., 2016) were analysed. Single-cell RNA sequencing (scRNA-seq) datasets representing baseline CD and fibrotic CD were obtained from GSE266546 (Li et al., 2024) and SCP2959 (Kong et al., 2025), respectively. In addition, the 16S rRNA sequencing dataset PRJNA398187 (Ryan et al., 2020) was included to analyse microbiome compositions. Detailed information regarding sample characteristics is summarised in Table 1.

**Table 1:** Summary of the transcriptomics and microbiome datasets used in this study.

Datasets	Technology	Group	Age, years (median [IQR])	Sex (%F/%M)	Tissue type	Sample type	References
Crohn’s disease and non-inflammatory bowel disease (transcriptomics)							
GSE230113	RNA-seq	CD (*n* = 10), NIBD (*n* = 10)	33.0 [26.0–42.2]	40.0%/60.0%	Colon	Biopsy	Wang et al. (2024)
GSE139179	RNA-seq	CD (*n* = 74), NIBD (*n* = 36)	37.0 [30.0–50.0]	73.6%/26.4%	Colon	Biopsy	Revilla et al. (2021)
GSE83687	RNA-seq	CD (*n* = 12), NIBD(*n* = 49)	62.0 [50.0–71.0]	55.7%/44.3%	Colon	Biopsy	Peters et al. (2017)
GSE266546	scRNA-seq	CD (*n* = 53), NIBD (*n* = 15)	44.0 [26.0–53.0]	65.6%/34.4%	Colon	Biopsy	Li et al. (2024)
Fibrotic Crohn’s disease and non-inflammatory bowel disease (transcriptomics)							
GSE123141	RNA-seq	Fibrosis CD (*n* = 9), NIBD (*n* = 9)	63.0 [46.0–70.5]	44.4%/55.6%	Colon	Biopsy	Dharmasiri et al. (2021)
GSE66207	RNA-seq	Fibrosis CD (*n* = 21), NIBD (*n* = 9)	45.0 [31.0–56.0]	60.6%/39.4%	Colon	Biopsy	Peck et al. (2015)
GSE67250	RNA-seq	Fibrosis CD (*n* = 3), NIBD (*n* = 3)	40.5 [31.3–50.0]	50.0%/50.0%	Colon	Colonic Resection	Sadler et al. (2016)
SCP2959	scRNA-seq	Fibrosis CD (*n* = 21), NIBD (*n* = 10)	50.0 [30.0–50.0]	45.2%/54.8%	Colon and Ileum	Colon Biopsy and Intestinal Resection	Kong et al. (2025)
Fibrotic Crohn’s disease, Crohn’s disease, and non-inflammatory bowel disease (microbiome)							
PRJNA398187	16S rRNA	CD (*n* = 38), Fibrosis CD (*n* = 11), NIBD (*n* = 31)	45 [34.0–59.0]	48.3%/51.7%	Colon	Biopsy	Ryan et al. (2020)

The table includes accession numbers for bulk RNA-seq, single-cell, and microbiome datasets for Baseline CD, Fibrosis CD, and Non-IBD (NIBD). It also provides key patient information, including the number of individuals in each group, median age, sex distribution, tissue type, and sample collection methods.

For reproducibility, datasets were identified through a systematic search of public repositories following PRISMA guidelines. Predefined keywords related to Crohn’s disease, intestinal fibrosis, colon tissue, and transcriptomic or microbiome profiling were used, and the full search strategy and dataset selection flow are detailed in the PRISMA diagrams (Supplementary Figures 1–3). Detailed information about the inclusion criteria and the dataset selection process is available in Supplementary Method 1.

### 2.2 Data preprocessing

Baseline CD and fibrosis CD bulk transcriptomic datasets were pre-processed as follows: gene identifiers in the raw data that corresponded to Entrez Gene IDs were mapped to their official HUGO Gene Nomenclature Committee (HGNC) gene symbols (Bruford et al., 2022). Genes lacking valid annotations were removed. Genes with zero counts across all samples were excluded, and to account for differences in library sizes, raw read counts were normalised to counts per million (CPM) (Luecken and Theis, 2019)^.^ The CPM values were then log₂-transformed as log₂(CPM + 1). Genes within the lowest 20th percentile of variance were removed to reduce the influence of minimally informative features. This conservative threshold retained approximately 80% of genes in each cohort while improving the signal-to-noise ratio prior to dataset integration. Further information on variance filtering is provided in Supplementary Table 2 and Supplementary Figure 4.

Microbiome data included patients with multiple biopsies; the sample with the highest sequencing depth was selected for downstream analysis to maximise data quality and minimise sparsity. Amplicon sequence variants (ASVs) were aggregated at the genus level, converted to relative abundances, and genera present in fewer than 20% of the samples were removed.

The data were subsequently transformed using the centered log-ratio (CLR) with a pseudo-count of 1 added to address zero inflation, followed by min-max normalisation. Alpha diversity, which reflects both the taxonomic richness and evenness within a sample, was quantified using the Shannon and Simpson indices. In contrast, beta diversity was assessed using Bray–Curtis dissimilarity and visualised by principal coordinates analysis (PCoA). The preprocessing pipeline was adapted from previously published microbiome workflows (Cassol et al., 2025; Lozupone and Knight, 2008; Hodgkiss and Acharjee, 2025; Ryan et al., 2020).

### 2.3 Batch correction and identification of differentially expressed genes

The baseline CD and fibrosis CD datasets were normalised and filtered separately, after which, datasets within each condition were harmonised using ComBat (Johnson et al., 2007) to account for batch effects arising from different sequencing platforms and experimental workflows. Sample-level metadata specifying batch origin and patient group were included as covariates, and the effectiveness of batch correction was assessed using principal component analysis (PCA) to visualise the reduction of batch-associated variation (Maćkiewicz and Ratajczak, 1993).

Differential gene expression (DGE) (Ritchie et al., 2015) analysis was then performed on the batch-corrected data to identify genes differentially expressed between baseline CD and NIBD, and between fibrosis CD and NIBD. Moderated *t*-statistics and *p*-values were computed using empirical Bayes moderation, and results were adjusted for multiple testing using the Benjamini–Hochberg false discovery rate (FDR) method (Benjamini and Hochberg, 1995).

### 2.4 Pathway enrichment analysis

Pathway enrichment analysis was performed to characterise the biological processes represented within the shared differentially expressed gene sets. Functional annotation using Gene Ontology (GO) (Ashburner et al., 2000) classified genes into biological process, molecular function, and cellular component categories to identify over-represented biological themes. Complementary pathway analyses were conducted using the Kyoto Encyclopaedia of Genes and Genomes (KEGG) (Kanehisa et al., 2019) and Reactome (Jassal et al., 2020) databases to identify enriched molecular pathways representing conserved immune, metabolic, and signalling processes represented within the transcriptional signatures.

To further capture higher-level biological programs, Hallmark pathway enrichment analysis was performed using the Molecular Signatures Database (MSigDB) (Liberzon et al., 2015). Hallmark pathways represent curated gene sets that summarise well-defined biological states or processes, such as inflammation, hypoxia, or epithelial-mesenchymal transition. By condensing redundant gene sets into 50 coherent biological programs, the Hallmark collection reduces noise and improves interpretability in high-dimensional transcriptomic analyses. Differentially expressed genes were mapped to MSigDB Hallmark gene sets for *Homo sapiens* (Subramanian et al., 2005). Over-representation analysis was conducted using a hypergeometric framework, comparing the gene sets of interest against a background universe of all expressed genes. Enrichment significance was assessed using two-sided tests, and *p*-values were adjusted for multiple testing using the Benjamini–Hochberg FDR correction. Pathways with an adjusted *p* < 0.05 were considered significantly enriched. Enrichment profiles were subsequently compared across disease groups (NIBD, baseline CD, and fibrosis CD) to identify biological programs exhibiting differential patterns across disease states.

### 2.5 Cell-type signature scoring

The scRNA-seq datasets from baseline CD and fibrosis CD patients were pre-processed individually, as described in Supplementary Method 2. Cell-type-specific gene signatures derived from scRNA-seq data were applied to bulk transcriptomic profiles for downstream analysis.

To explore the pathway-level activity changes in bulk transcriptomics data, we performed Gene Set Variation Analysis (GSVA) (Hänzelmann et al., 2013). This non-parametric unsupervised method estimates gene set enrichment scores per sample, enabling assessment of pathway activity variation across conditions. Cell-type-specific gene sets derived from scRNA-seq marker genes for both baseline CD and fibrosis CD were used as input to GSVA to estimate the relative enrichment of each cell-type-associated transcriptional signature across bulk samples, enabling comparison of these signatures across disease states (Santos and Acharjee, 2026).

### 2.6 Model development and predictive modelling

In this study, machine learning models were trained on transcriptomic data to distinguish baseline CD from NIBD patients. Features were defined as the intersection of DEGs identified in the baseline and fibrosis CD. Feature selection was then performed on this gene set using baseline CD data, and the selected genes were subsequently applied to the fibrosis CD dataset to evaluate predictive performance (Philip et al., 2026).

For the microbiome data, models were similarly trained on baseline CD to identify genera consistently selected across models. These genera were then used to predict fibrosis CD. In parallel, models were independently applied to fibrosis CD data to identify consistently selected genera, with the overlap between baseline and fibrosis CD defined as shared taxa.

The three machine learning models used were: eXtreme Gradient Boosting (XGBoost) (Chen and Guestrin, 2016), Random Forest (Breiman, 2001), and Least Absolute Shrinkage and Selection Operator (LASSO) (Tibshiranit, 1996). XGBoost is an ensemble algorithm that iteratively refines predictions with built-in regularisation to prevent overfitting. At the same time, Random Forest constructs multiple decision trees from random feature subsets, reducing variance and improving model stability. For these tree-based models, Kursa et al. (2010) feature selection was applied to identify statistically significant features by comparing the actual feature importance with that of randomised “shadow” features.

LASSO was selected for its ability to perform both classification and feature selection through L1 regularisation. In binary classification, it uses a regularisation parameter, C, the inverse of *λ* (C = 1/λ), where higher C values reduce regularisation and retain more features, while lower values increase regularisation and shrink non-informative coefficients toward zero.

To identify features and optimise model performance, a nested cross-validation (CV) framework was implemented. A 10-fold outer stratified CV was used to evaluate model generalisability, while an inner 5-fold CV was used for hyperparameter optimisation (Acharjee et al., 2020). For each outer training set, optimal hyperparameters were determined using Optuna (Akiba et al., 2019; Philip et al., 2025), a Bayesian optimisation framework that maximises the mean area under the receiver operating characteristic curve (ROC-AUC) across inner folds for transcriptomics data. For microbiome data, hyperparameters were selected using GridSearchCV (Liashchynskyi and Liashchynskyi, 2019) within the same inner cross-validation framework.

Once the optimal hyperparameters were obtained, a final model was retrained on the entire outer training fold and evaluated on the corresponding test fold. Performance was assessed using multiple complementary metrics, including accuracy, precision, recall, F1-score, AUC, and specificity.

Following model development and internal evaluation, the previously trained baseline CD models were used for predictive modelling on the fibrosis CD datasets. For both the transcriptomic and microbiome analysis, the genes consistently identified by XGBoost, Random Forest, and LASSO were extracted from the fibrosis CD dataset and used as input features for the previously trained baseline CD models. The models were applied directly to the fibrosis CD samples using the optimal hyperparameters identified during model development, and predictive performance was evaluated. A detailed machine learning workflow is available in Supplementary Figure 5.

Additionally, to evaluate whether heterogeneity within the 21 NIBD samples in the fibrosis transcriptomics cohort influenced model performance, a sensitivity analysis was performed. The fibrosis samples were retained unchanged, while the NIBD controls were stratified into three biologically relevant subgroups: The neoplastic group (adenomas and polyps; NIBD *n* = 12), the inflammatory group (diverticulitis; NIBD *n* = 5), and the non-inflammatory group (colonic inertia and related benign conditions; NIBD *n* = 4) (Supplementary Method 3). The XGBoost, Random Forest, and LASSO models trained on the baseline CD cohort were then applied separately to each NIBD subgroup in combination with the fibrosis samples. Predictive performance was assessed using the same metrics as the primary analysis.

### 2.7 Feature selection and correlation networks for Transcriptomics

Candidate genes were identified as the intersection of the features derived independently from Random Forest, XGBoost, and LASSO. Only genes consistently prioritised across all three models were retained for downstream analysis to identify features consistently selected across models.

To further refine the genes identified through machine learning and assess how their behaviour changes across disease states, a likelihood ratio test (LRT) (Birkes, 2006) was used to identify genes that vary across disease stages by comparing models with and without the disease group as a factor. To examine the changes, pairwise comparisons were derived from baseline CD vs. NIBD and fibrosis CD vs. baseline CD. Genes were retained if they had an adjusted *p*-value < 0.01 and a log2-fold change >1.5 (in either direction) in at least one comparison. A matrix of the log2 fold changes was then created to represent stage-associated changes across disease groups. This was visualised in the form of a heatmap, with colour indicating the direction and magnitude of change. Statistical significance for each gene was added using asterisks based on the adjusted *p*-values.

To characterise the transcriptional coordination among the significant log-fold change genes, a gene–gene correlation network was constructed using merged, normalised expression data across all samples. Pairwise Spearman correlations were computed between all gene pairs. The *p*-values were adjusted using the Benjamini–Hochberg method, and edges were retained based on predefined thresholds for correlation magnitude (|*ρ*| ≥ 0.6) and FDR ≤ 0.05. An undirected weighted network was constructed using significant correlations, with edge weights defined as the absolute correlation coefficient (|ρ|). Community structure was identified using the Louvain algorithm for modularity optimisation. Subsequent analyses focused primarily on the largest connected component of the network.

Node strength, defined as the sum of absolute edge weights connected to each gene (*Σ*|ρ|), was used as a measure of network centrality. Hub genes were defined using a statistical threshold (mean + 1 SD) to identify highly connected nodes. To characterise the biological themes represented within each module, pathway coverage analysis was performed using MSigDB Hallmark, GO Biological Process, KEGG, and Reactome gene sets. Given the small module sizes, this analysis was descriptive rather than inferential.

### 2.8 Knowledge-based integration of transcriptomic and microbiome findings

Since the transcriptomic and microbiome datasets were obtained from different patient cohorts, not from matched samples, we could not perform direct multi-omics integration. Hence, a knowledge-based (qualitative) integration approach was applied, similar to Bisht et al.(Bisht et al., 2021). This approach allowed biological interpretation of transcriptomic and microbiome data by integrating the observed host molecular changes with the microbiome data generated in this study and supported by the current understanding of CD pathogenesis. The proposed framework for knowledge-based integration is based on the well-established multi-omics integration strategies that combine multiple omics layers to generate biologically plausible mechanistic hypotheses even when datasets cannot be directly integrated at the individual sample level.

### 2.9 Synthetic data generation and modelling framework

In this work, we initially generated synthetic RNA-seq profiles representing fibrotic CD (Table 1) that capture the molecular markers and progression patterns of fibrotic CD by combining SPsimSeq (Assefa et al., 2020) with biologically based LLM-guided prompting. Instead of using abstract text-based predictions, we produced simulated trajectories that reflect reasonable disease dynamics by linking LLM argumentation to actual gene-expression distributions and carefully selected fibrotic CD-specific biology. This framework provides a controlled approach to investigating biomarker behaviour and fibrosis-related mechanisms in settings where datasets are limited or fragmented.

Synthetic RNA-seq profiles were generated using a hybrid approach combining rule-based prompting of a generative language model with data simulation. The main goal was to create transcriptome profiles unique to fibrosis CD that represent biological characteristics associated with intestinal fibrosis as well as patient-level variables. This was accomplished by first generating synthetic bulk RNA-seq data using normalised CPM matrices using SPsimSeq, which included metadata factors such as age, sex, and disease label. We applied OpenAI’s GPT-4.1-mini model to conditionally create gene expression profiles using patient metadata and biological rules obtained from the literature (Supplementary Figure 6A).

In a second analysis, we examined the impact of adding a new group of genes linked to fibrosis from the scientific literature to the dataset. To add gene expression patterns consistent with intestinal fibrosis and inflammatory activity, the model was guided using structured, rule-based instructions. Covariate-conditioned modulation was applied to a curated panel of 40 fibrosis-associated genes that were compiled from previously published research (Verstockt and Cleynen, 2016; Park et al., 2023; Lovisa and Vetrano, 2024; Acharjee et al., 2025). The prompt was directly encoded with biological rules and metadata for each synthetic sample, including the expected direction of differential expression, the inflammatory state, and the severity of fibrosis. To create the final covariate-conditioned expression profiles, the gene-specific multiplicative scaling factors created by GPT 4.1 mini were applied to the CPM values provided by SPsimSeq. This hybrid method introduced biologically significant variance consistent with the pathogenesis of fibrosis CD while maintaining statistical realism. The LLM prompt we have used in this analysis is available in Supplementary Method 4.

The combined SPsimSeq-LLM framework generated a larger CPM matrix and updated metadata that included fibrosis severity categories and covariate data. The produced datasets were utilised for differential expression analysis to discover gene-level patterns associated with fibrosis CD and examine the effect of synthetic covariate-conditioning on simulated transcriptomic patterns across diseases. (Supplementary Figure 6B).

### 2.10 Statistical and predictive validation of the SPsimSeq–LLM framework

To evaluate whether the synthetic datasets preserved the statistical characteristics of the original gene expression data. First, we examined the overall expression distributions of the original, SPsimSeq-generated, and LLM-generated datasets. Both augmentation techniques were successful in maintaining the global expression profile of the original dataset, as evidenced by the density distributions’ very similar forms.

We applied a Kullback–Leibler (KL) (Zhang et al., 2025) divergence analysis between the gene expression patterns of CD and control to further measure distributional similarities. Histogram-based density estimation with pseudo-count regularisation was used to transform gene expression values into probability distributions, and density plots and histograms were used to summarise gene-wise KL divergence values. Both synthetic datasets accurately maintained the statistical characteristics of the original gene expression distributions without introducing significant distributional bias, as evidenced by the highly comparable KL divergence value distributions between the original, SPsimSeq-generated, and LLM-generated datasets.

Next, we used Pearson correlation heatmaps to analyse the sample-to-sample correlation structure. The correlation patterns found in both synthetic datasets were quite similar to the actual data, indicating that the augmentation techniques maintained the global covariance structure of gene expression and the underlying linkages between samples.

Finally, we used a Random Forest classifier to assess the usefulness of the original, SPsimSeq-generated, and LLM-generated datasets for downstream machine learning classification. Model performance was assessed using repeated stratified 5-fold cross-validation (10 repeats). To limit data leaking from the test data, the top 200 differentially expressed genes were chosen as predictive features after differential expression analysis was carried out inside each training fold to rank genes. The chosen features were used to train a Random Forest model with 500 decision trees, which was then assessed on the matching test fold. AUC, accuracy, F1-score, sensitivity, and specificity were used to evaluate performance. The mean and 95% empirical confidence intervals for each cross-validation fold were used to summarise the final performance.

## 3 Results

### 3.1 Demographic characteristics

The study included multiple publicly available cohorts spanning bulk RNA-seq, single-cell RNA-seq, and 16S rRNA sequencing, including samples from individuals with NIBD, baseline CD, and fibrosis CD (Table 1). All transcriptomic datasets were derived from intestinal tissue, primarily colonic biopsies, with a subset of fibrosis CD samples obtained from surgical resections and the ileum. Control samples from all datasets were grouped under NIBD and included healthy individuals, adenoma cases, diverticulitis cases, and other non-inflammatory controls.

Bulk RNA-seq datasets had median ages ranging from 33.0 (26.0–42.2) to 63.0 (46.0–70.5) years. Sex distributions were similar across cohorts, with female representation ranging from 40.0 to 73.6%. Single-cell RNA-seq datasets comprised baseline, fibrosis CD, and NIBD samples, with median ages ranging from 44.0 (26.0–53.0) to 50.0 (30.0–50.0) years, and balanced sex distributions between disease groups.

A microbiome cohort based on 16S rRNA sequencing was also included, comprising baseline CD and fibrosis CD patients. All samples were derived from colonic biopsies, with a median age of 45.0 (34.0–59.0) years, and female representation of 48.3% across the cohort.

### 3.2 Batch correction and differential gene expression analysis

Bulk RNA-seq datasets were jointly analysed, with comparisons performed between baseline CD versus NIBD and fibrosis CD versus NIBD. After filtering out genes with low expression and low variability from both the baseline CD and fibrosis CD datasets, the remaining genes were aligned across datasets before merging, and batch effects were corrected using ComBat.

**Figure 2:**
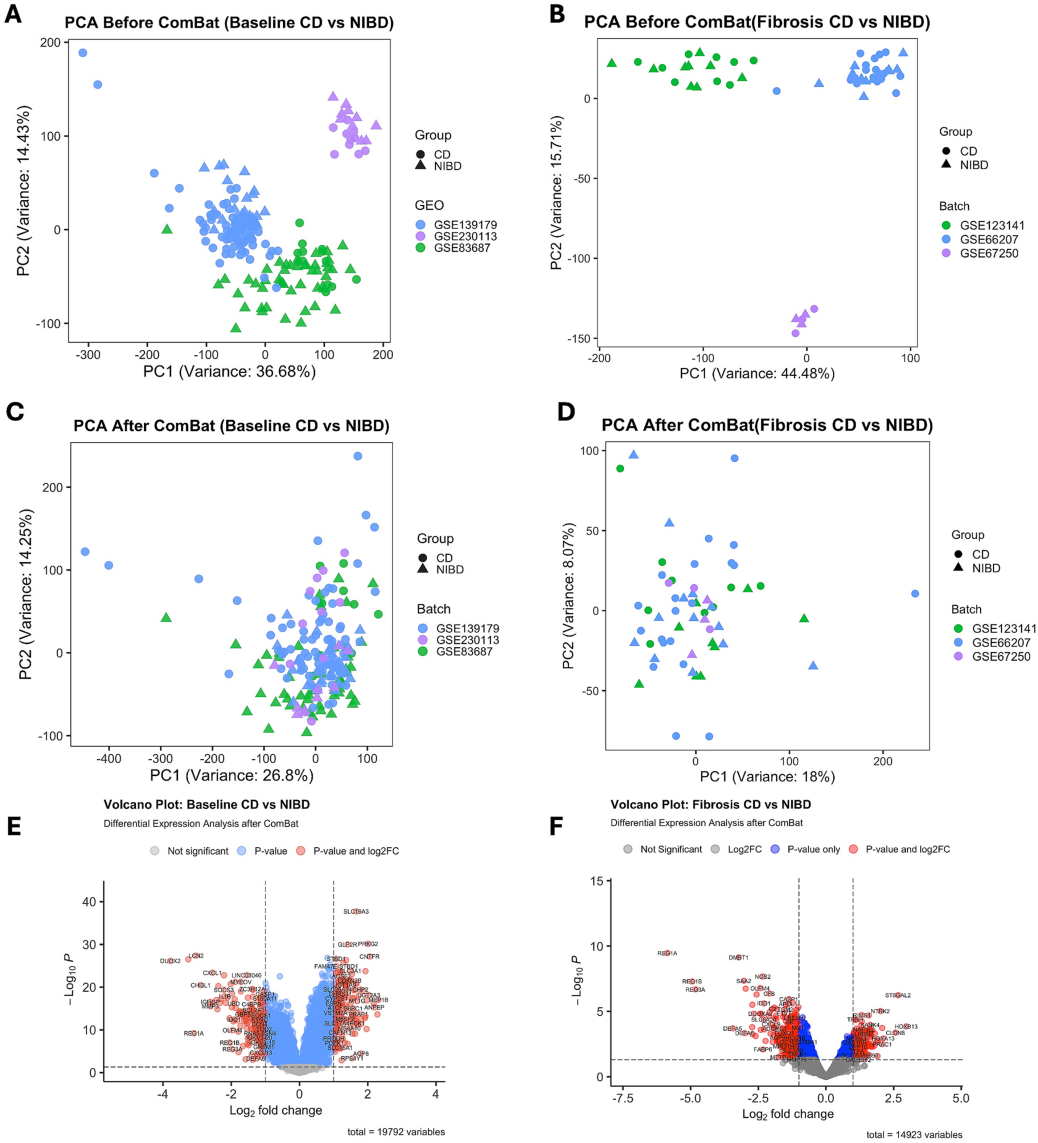
Batch correction and differential expression workflow across disease states. PCA and differential gene expression analyses were performed to evaluate batch effects and transcriptional differences between disease groups. **(A,B)** PCA plots show batch effects before ComBat for Baseline CD vs. NIBD and Fibrosis CD vs. NIBD. **(C,D)** PCA plots after ComBat batch correction for Baseline CD vs. NIBD and Fibrosis CD vs. NIBD. Samples are coloured by the GEO dataset and shaped by clinical group, with variance explained indicated on the axes. **(E,F)** Volcano plots summarise differential expression results from batch-corrected data, with significant genes highlighted based on combined statistical and fold-change thresholds.

Before batch correction, PCA showed strong clustering by study of origin rather than disease group, indicating substantial dataset-specific effects (Figures 2A,B). Following batch correction, PCA showed reduced clustering by study of origin, with samples from different studies overlapping in the reduced-dimensional space (Figures 2C,D). The variance explained by the leading principal components decreased after correction, consistent with the reduction of technical variability while preserving disease-related transcriptional structure.

Differential expression analysis of the corrected datasets identified disease-associated genes. Volcano plots illustrate the distribution of effect sizes and statistical significance for baseline CD versus NIBD and fibrosis CD versus NIBD (Figures 2E,F).

In total, 456 significant DEGs (adjusted *p*-value < 0.05 and log2FC ≥ 2) were identified for baseline CD vs. NIBD, and 432 genes (adjusted *p-*value < 0.05 and log2FC ≥ 1) for fibrosis CD vs. NIBD. A less stringent log2FC threshold was applied for fibrosis CD because transcriptomic changes were generally of smaller magnitude than those observed at baseline. Using a threshold of log2FC ≥ 2 resulted in too few shared genes for downstream analysis. Therefore, the lower threshold was selected to retain biologically relevant genes while providing a sufficient feature set for subsequent machine learning. We then focused on the intersection of the baseline CD and fibrosis CD gene sets, resulting in 94 shared genes across both cohorts.

### 3.3 Pathway enrichment

**Figure 3:**
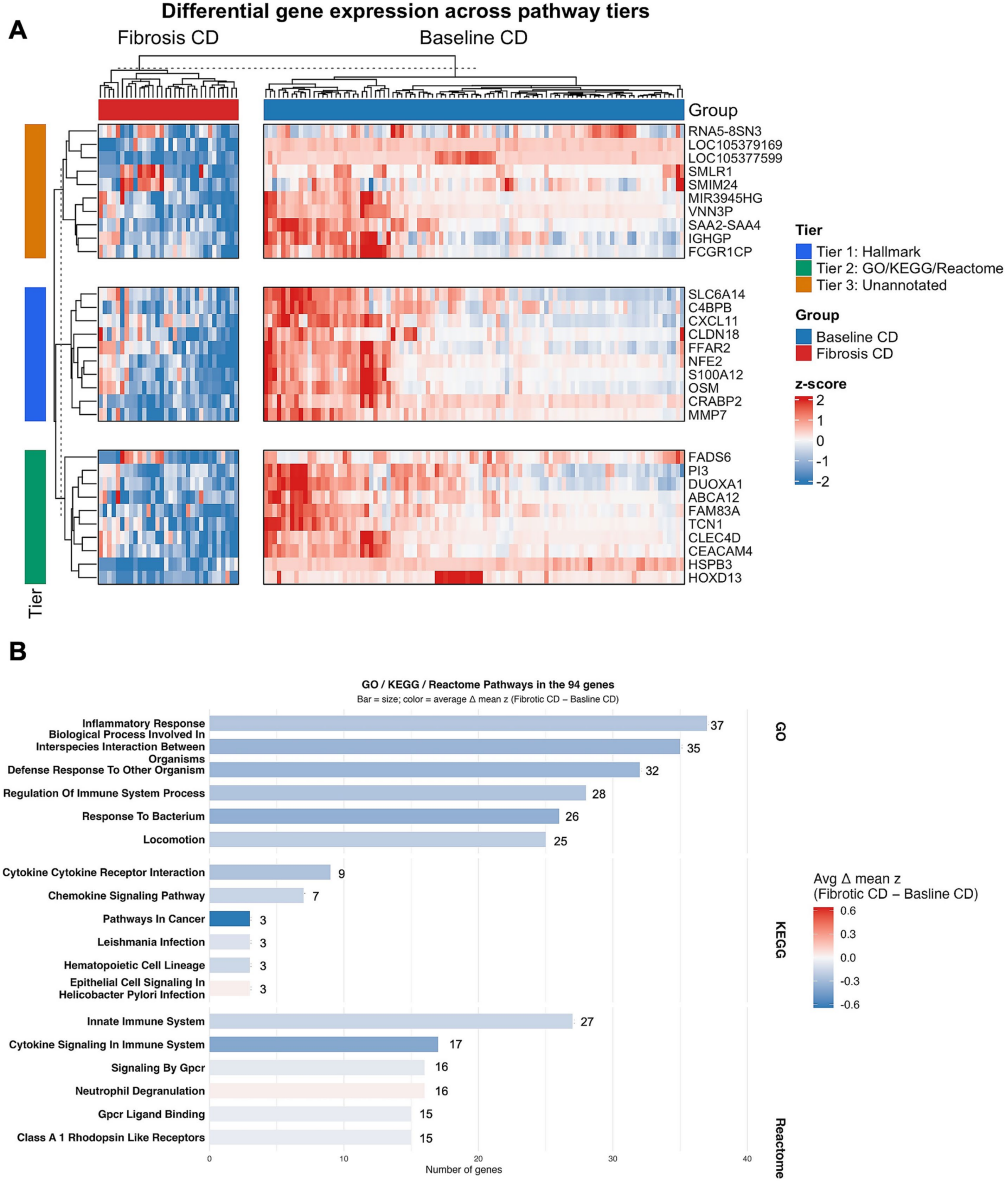
Differential gene expression and pathway enrichment across pathway tiers in Crohn’s disease **(A)** Heatmaps display scaled (*z*-score) expression of differentially expressed genes between Crohn’s disease (CD) and fibrosis CD, organised into three pathway tiers: Tier 1, Hallmark pathways; Tier 2, GO/KEGG/Reactome-annotated genes; and Tier 3, unannotated genes. Samples are clustered hierarchically, and group annotations indicate Baseline CD and Fibrosis CD status. **(B)** The bar plot summarises enriched GO, KEGG, and Reactome pathways identified among the 94 genes, with bar length representing the number of genes per pathway and colour indicating the average difference in expression.

Pathway enrichment analysis was performed on the set of 94 differentially expressed genes. Genes were organised into three tiers based on annotation status, and hierarchical clustering revealed coordinated expression changes across samples, with consistent separation between baseline CD and fibrosis CD groups (Figure 3A). Differential expression patterns were observed across all pathway tiers, including Hallmark pathways, GO/KEGG/Reactome-annotated genes, and unannotated genes, suggesting widespread transcriptional differences associated with fibrosis CD.

Enrichment analysis of GO, KEGG, and Reactome terms identified pathways largely related to immune and inflammatory processes, including inflammatory response, regulation of immune system processes, host defence, cytokine and chemokine signalling, innate immune system activity, and neutrophil degranulation. Notably, the enrichment of immune-related pathways across the shared gene set suggests persistence of inflammatory programs across both baseline and fibrosis CD (Figure 3B).

### 3.4 Model training and prediction modelling for Transcriptomics

**Figure 4:**
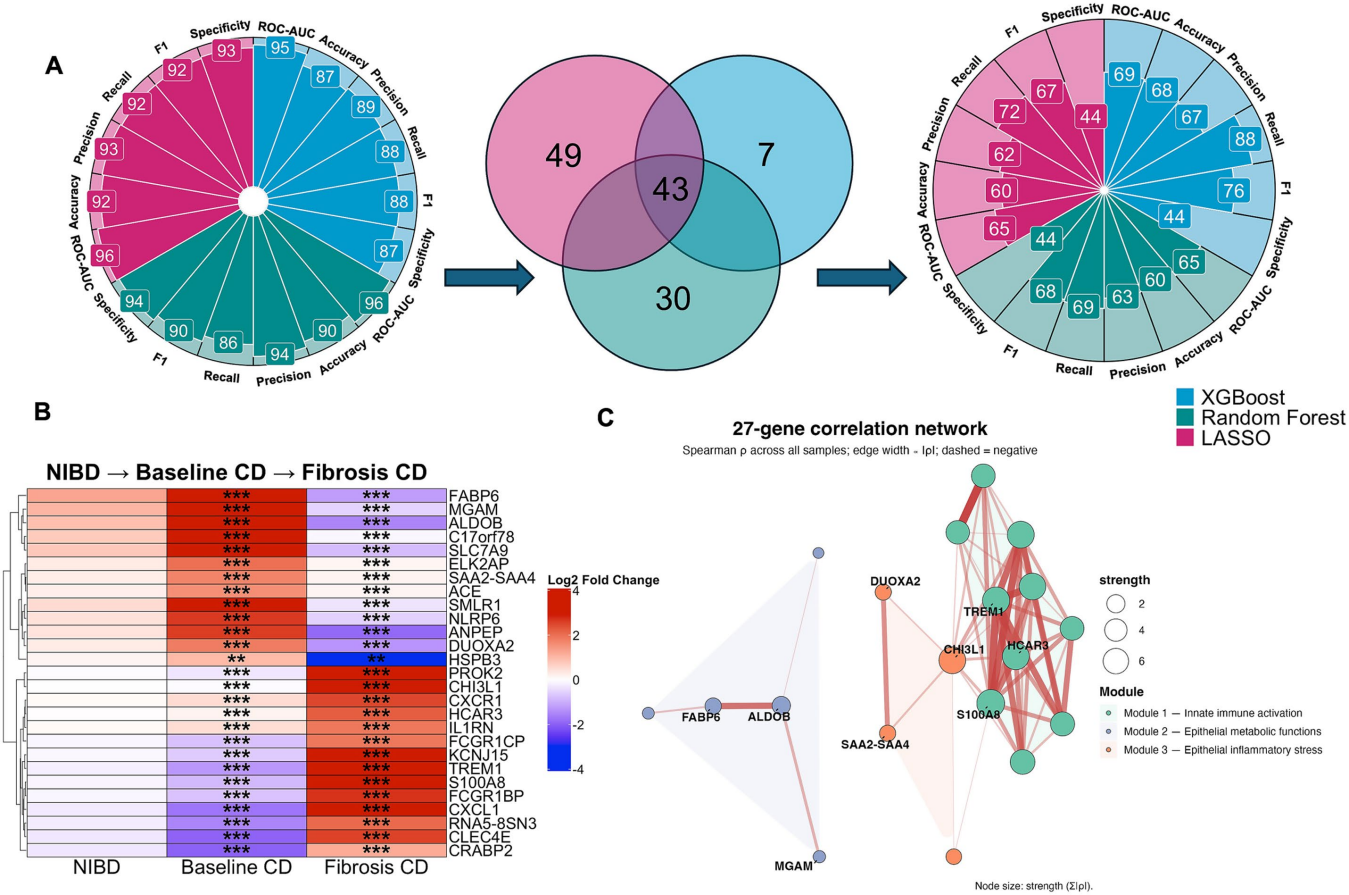
Genes identified by machine learning show progressive expression changes and network remodelling in Crohn’s disease to fibrosis progression **(A)**. Three machine learning models, XGBoost, Random Forest, and LASSO, were trained on baseline CD samples, resulting in the identification of 43 overlapping genes across all three models, as shown in the Venn diagram. Genes identified as important in the baseline CD dataset were extracted from the fibrosis CD dataset and used for fibrosis CD prediction. The overall model performance is summarised in the circular bar plot **(B)**. Heatmap showing log₂ fold-change expression of the 27 genes that had significant changes in expression across Non-IBD (NIBD), Baseline CD, and Fibrotic CD, illustrating progressive transcriptional shifts with disease severity **(C)**. The correlation network of the 27 genes highlights genes separated into three modules. Nodes represent genes, with size proportional to connectivity and edge thickness indicating correlation strength; colours denote functional modules.

Three machine learning models were employed in this study: XGBoost, Random Forest, and LASSO. The models were trained to distinguish baseline CD from NIBD using the 94 DEGs shared between baseline and fibrosis CD, selected to focus on common transcriptional signals. Hyperparameter optimisation was performed using Bayesian optimisation, and feature selection was carried out using Boruta for the tree-based models, while LASSO performed feature selection as part of the model. Based on collective performance metrics (Figure 4A), 50 genes were identified as important by XGBoost, 73 by Random Forest, and 92 by LASSO. Overall, 43 genes overlapped across all three models and were selected for further analysis. Random Forest and LASSO showed the highest performance with an AUC of 0.96 (0.94–0.98). Random Forest had an accuracy of 0.90 (0.86–0.93), while LASSO achieved an accuracy of 0.92 (0.89–0.96). XGBoost followed closely, with an AUC of 0.95 (0.93–0.98) and an accuracy of 0.87 (0.82–0.93).

The 43 intersecting genes were subsequently evaluated in the fibrosis CD datasets. The previously trained baseline CD models, using their optimal hyperparameters, were applied for prediction, and performance metrics were recorded. XGBoost showed moderate performance, achieving an AUC of 0.69 (0.54–0.77) with 0.68 (0.54–0.81) accuracy in distinguishing between fibrosis and NIBD patients. Random Forest and LASSO followed, both achieving an AUC of 0.65 (0.49–0.79) and an accuracy of 0.60 (0.47–0.72). Additional performance metrics and the optimal hyperparameters for the machine learning models are reported in (Supplementary Tables 3, 4).

To determine whether the composition of the NIBD cohort influenced classifier performance, the controls were stratified into neoplastic, inflammatory, and non-inflammatory subgroups and analysed separately. When evaluated against the neoplastic subgroup, model performance slightly improved compared to the prediction models that had the full NIBD cohort. XGBoost achieved an AUC of 0.75 (0.53–0.83) compared with 0.69 (0.54–0.77) in the combined fibrosis NIBD analysis, while Random Forest and LASSO achieved AUCs of 0.66 (0.49–0.83) and 0.69 (0.52–0.85), respectively, compared with 0.65 for both models in the full cohort.

Similar performance was observed for the inflammatory subgroup with AUCs of 0.68 (0.41–0.93), 0.62 (0.38–0.81), and 0.72 (0.41–0.95) for XGBoost, Random Forest, and LASSO, respectively. In contrast, performance was lower with the non-inflammatory samples, with AUCs of 0.45 (0.39–0.92), 0.47 (0.08–0.89), and 0.39 (0.07–0.75) across the three models. (Supplementary Table 5).

### 3.5 Characterising significant log-change genes across disease stages

Intersecting the features across XGBoost, Random Forest, and LASSO models yielded a 43-gene set. Ranking these genes by log-fold change across disease groups revealed 27 genes exhibiting significant log-fold change from NIBD to baseline CD and further to fibrosis CD. The heatmap representing the log2-fold change (Figure 4B) reveals two distinct expression patterns. A group of genes, including MGAM, ALDOB, FABP6, SLC7A9, ANPEP, and DUOXA2, among others, was significantly upregulated in baseline CD relative to NIBD and downregulated in fibrosis CD. In contrast, another set of genes, which included CHI3L1, CXCL1, S100A8, TREM1, and IL1RN, showed progressively higher expression across the disease groups, with the highest expression observed in fibrosis CD.

Notably, some genes, such as SAA2-SAA4, were already elevated in baseline CD and remained dysregulated in fibrosis CD (Supplementary Table 6). Network analysis of the 27 selected genes identified 19 genes forming a connected component under the specified correlation (|*ρ*| threshold) and false discovery rate (FDR ≤ 0.05) criteria.

These genes were used to construct an undirected weighted gene–gene correlation network based on Spearman correlation across all samples (Figure 4C). Community detection using the Louvain algorithm revealed three principal modules, comprising 10, 5, and 4 genes, respectively. The distribution of node strength (defined as the sum of absolute edge weights) was non-uniform (mea*n* = 5.11; SD = 2.32), indicating heterogeneous connectivity within the network and the presence of highly connected nodes. Applying a statistical threshold of mean + 1 SD (strength ≥ 7.44) identified S100A8 as the only gene meeting criteria for a highly connected hub. Module 1 comprised 10 genes: S100A8, TREM1, HCAR3, CXCR1, CLEC4E, CXCL1, KCNJ15, PROK2, FCGR1BP, and FCGR1CP. Module 2 comprised 5 genes: ALDOB, FABP6, MGAM, SLC7A9, and ANPEP. Finally, Module 3 comprised 4 genes: CHI3L1, SAA2-SAA4, DUOXA2, and IL1RN (Supplementary Table 7).

To characterise the biological themes represented in each module, pathway coverage analysis was performed using MSigDB Hallmark, GO Biological Process, KEGG, and Reactome gene sets. Given the small module sizes, this analysis was descriptive rather than inferential. Module 1 genes were enriched for pathways related to innate immune activation, neutrophil degranulation, and inflammatory signalling. Module 2 was associated with epithelial metabolic processes, including carbohydrate metabolism and nutrient absorption.

Module 3 showed enrichment for epithelial stress responses, acute-phase signalling, and reactive oxygen species metabolism. Additionally, the module-specific genes from the bulk RNA seq analyses were visualised in the corresponding baseline CD and fibrosis CD scRNA-seq datasets as feature plots to determine their cellular localisation and expression patterns across annotated cell populations (Supplementary Figures 7A,B).

### 3.6 Cell-type signature enrichment analysis

The Gene Set Variation Analysis (GSVA) revealed distinct cell-type-associated transcriptional signatures across disease stages. Using DEGs identified in baseline CD (*n* = 456) and fibrosis CD (*n* = 432) and focusing on the 43 genes identified from machine learning, GSVA identified 11 significant cell types in baseline CD (FDR < 0.05) and 26 significant cell types in fibrosis CD (FDR < 0.01).

These results indicate that shared genes function in distinct cellular contexts depending on disease stage. For instance, LCN2 was enriched in early goblet and proliferating goblet cells in baseline CD, whereas in fibrosis CD, it was observed in more differentiated epithelial populations, including Colonocytes-LGALS4-hi and Stem-like cells-OLFM4-hi. Similarly, MMP3 showed enrichment in non-inflammatory fibroblast populations in baseline CD but showed enrichment in inflammatory fibroblasts and stromal-associated populations, including telocytes, in fibrosis CD (Supplementary Table 8; Supplementary Figures 8, 9).

### 3.7 Microbiome pre-processing

After including only active, inflamed biopsies, a total of 80 samples and 160 genera were retained, which were further reduced to 40 after removing low-abundance genera. After normalisation and transformation, alpha and beta diversity were calculated.

**Figure 5:**
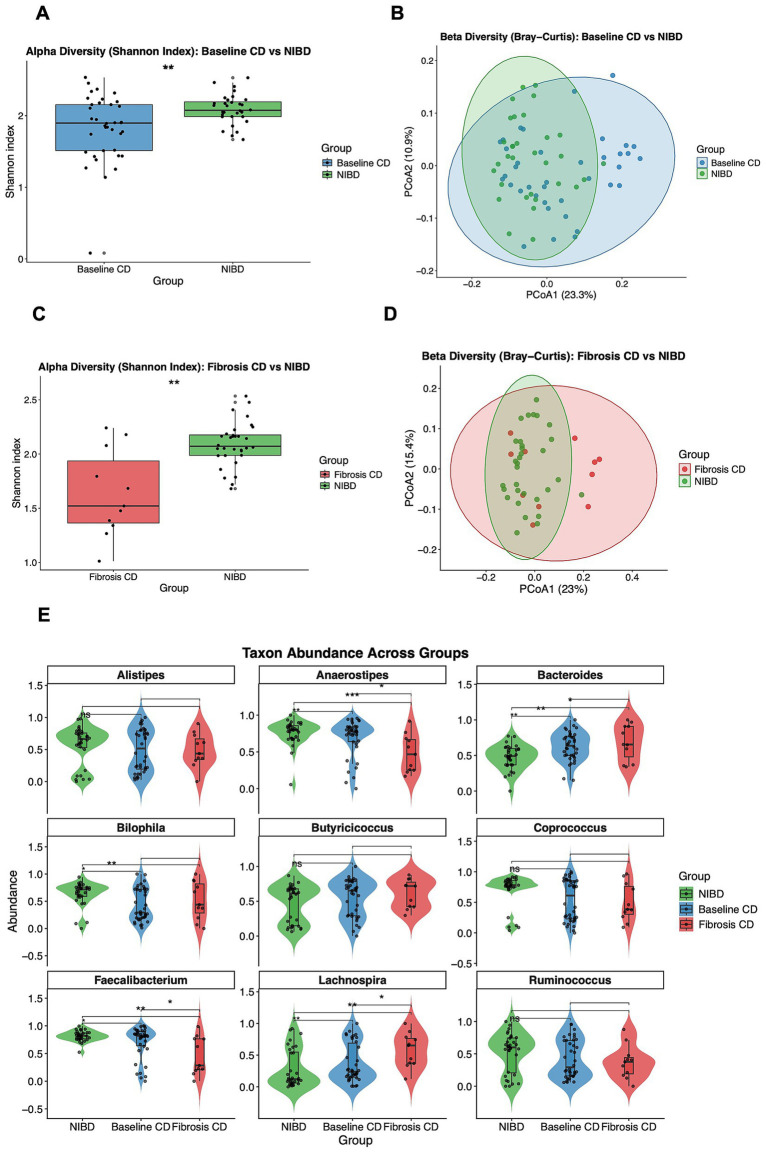
Alpha and beta diversity analyses of gut microbiome composition in Crohn’s disease (CD) subgroups. **(A)** Shannon diversity index comparing baseline CD patients and NIBD, showing significantly reduced alpha diversity in CD (Wilcoxon rank-sum test, *p* = 0.007). **(B)** Principal coordinates analysis (PCoA) based on Bray–Curtis dissimilarity demonstrates partial separation between baseline CD patients and NIBD. **(C)** Shannon diversity index comparing fibrosis CD patients and NIBD, indicating significantly lower microbial diversity in fibrosis CD (Wilcoxon rank-sum test, *p* = 0.0027). **(D)** Bray-Curtis PCoA illustrating altered community structure in fibrosis CD relative to NIBD. Ellipses represent 95% confidence intervals around group centroids. **(E)** Genus-level abundance of the nine taxa shared between baseline CD and fibrosis CD across the three groups. Statistical significance was assessed using the Kruskal–Wallis test.

When comparing microbial diversity across disease stages, both baseline CD and fibrosis CD exhibited significantly lower alpha diversity (Shannon index) compared to NIBD (baseline CD: *p-*value = 0.007; fibrosis CD: *p*-value = 0.002) (Figures 5A,C).

Consistently, beta diversity analysis demonstrated partial clustering of CD samples relative to NIBD in both stages, indicating differences in overall microbial community composition, although with some overlap between groups (Figures 5B,D).

### 3.8 Model training and prediction modelling for microbiome

The same models used for the transcriptomic datasets were also applied to the microbiome data. Model performance was evaluated using nested stratified 10-fold cross-validation, with GridSearchCV and an inner stratified 5-fold cross-validation for hyperparameter tuning. The performance was reported as mean values with 95% confidence intervals derived from the outer-fold scores.

For baseline CD classification (baseline CD versus NIBD), the LASSO model selected 27 non-zero features. In comparison, Random Forest and XGBoost with Boruta selected 11 features each, achieving AUCs of 0.80 (0.67–0.94), 0.75 (0.56–0.92), and 0.70 (0.50–0.88), respectively. A total of 8 genera were consistently selected across all three machine learning models, including *Bilophila, Bacteroides, Veillonella, Dialister, Bifidobacterium, Butyricicoccus, Ruminococcus, Sutterella.*

For fibrosis CD, the same models were used to distinguish NIBD from fibrotic CD patients. LASSO identified 8 genera, while Random Forest and XGBoost identified 11 and 10, respectively, with AUCs of 0.84 (0.64–1.00), 0.79 (0.63–0.95), and 0.75 (0.55–0.95), respectively. The models identified 7 genera shared across all models, including *Coprococcus, Faecalibacterium, Prevotella, Bilophila, Ruminococcus, Lachnospira, Phascolarctobacterium.*

The 29 genera identified as important features in baseline CD were subsequently used to train the models for fibrosis CD, to assess whether microbial signatures derived from early disease stages can predict fibrosis (Supplementary Figure 10). AUCs for LASSO, Random Forest, and XGBoost were 0.84 (0.64–1.00), 0.83 (0.58–1.00), and 0.81 (0.57–1.00), respectively. Additional performance metrics and optimised hyperparameters are reported in Supplementary Tables 9, 10.

In parallel, the 9 genera identified as overlapping between baseline CD and fibrosis CD were further analysed for relative abundance across NIBD, baseline CD, and fibrosis CD, revealing significant differences in key taxa, including *Alistipes, Anaerostipes, Bacteroides, Bilophila, Butyricicoccus, Coprococcus, Lachnospira, Ruminococcus, and Faecalibacterium.* (Figure 5E).

### 3.9 Microbiome and transcriptome knowledge-based integration

**Figure 6:**
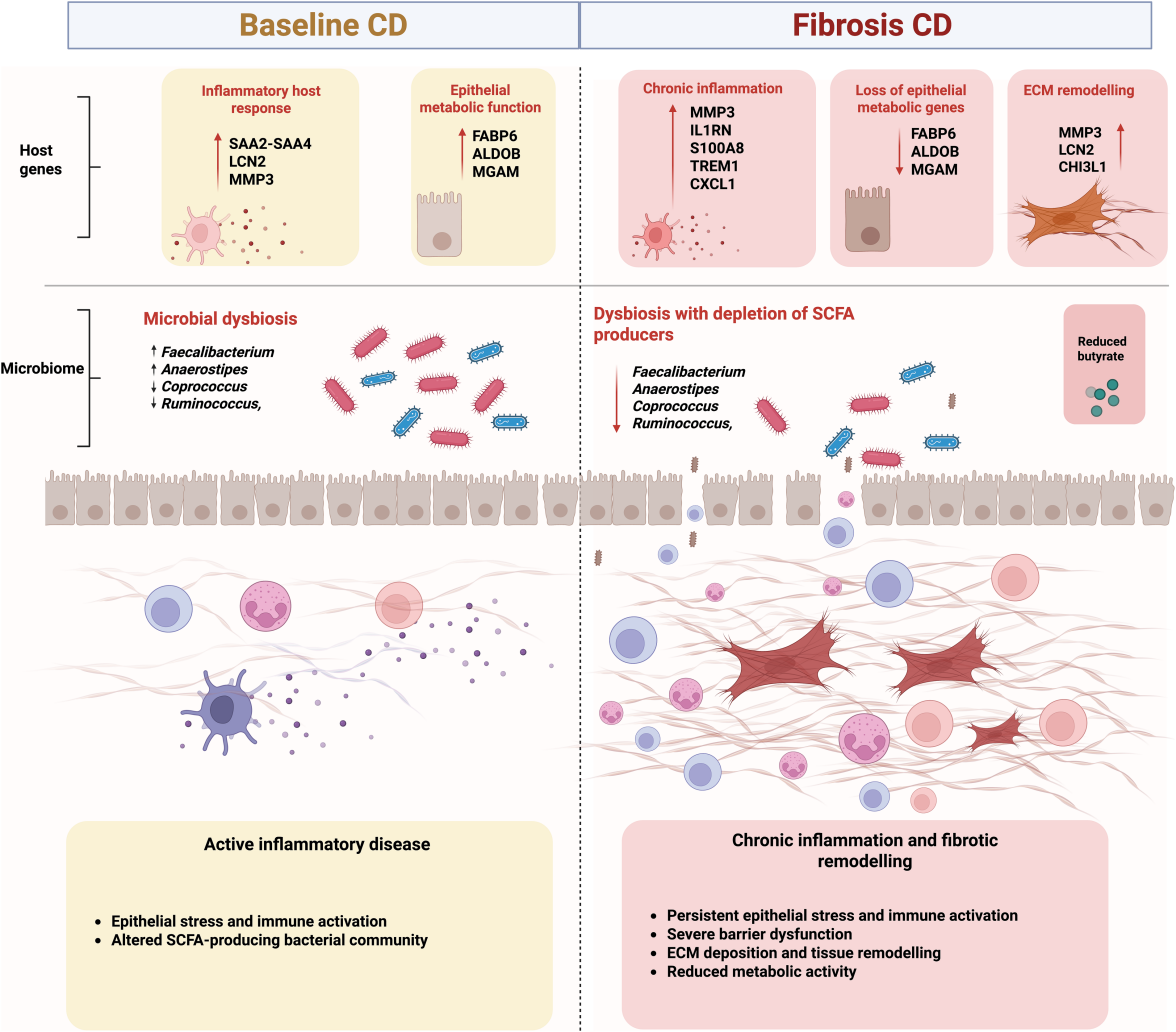
Knowledge-based integration of host transcriptomic and microbiome alterations between baseline and CD pathogenesis. This knowledge base approach integrates the transcriptomic and microbiome findings identified in the present study to illustrate potential interactions between host inflammatory responses, epithelial dysfunction, microbial dysbiosis, and fibrotic remodelling.

The qualitative integration approach illustrates that baseline CD is characterised by activation of inflammatory response genes, including SAA2–SAA4, LCN2. Meanwhile, microbiome analysis demonstrated depletion of some SCFA-producing genera, including *Coprococcus* and *Ruminococcus*, indicating dysbiosis that is already established during the inflammatory stage of disease (Figure 6).

In fibrotic CD, the transcriptomic profile shifted towards chronic inflammation, wound healing, and extracellular matrix remodelling, characterised by increased expression of CHI3L1, IL1RN, LCN2, and MMP3, and reduced expression of epithelial metabolic genes, including FABP6, ALDOB, and MGAM. These host alterations associated with persistent depletion of SCFA-producing bacteria. Together, these observations support a knowledge-based approach in which persistent inflammation, epithelial metabolic dysfunction, and microbiome dysbiosis coexist within a microenvironment that is associated with fibrotic remodelling in CD.

### 3.10 Synthetic data generation using LLM

In performance-vs-augmentation curves, an elbow point is the level of augmentation where fractional progress slows down significantly, indicating a realistic optimum rather than adding more synthetic samples with little extra advantage (Supplementary Figure 11). To explore the effect of synthetic data augmentation on model performance, we trained a *RandomForestClassifier* with different levels of synthetic sample addition and evaluated performance using a *RepeatedStratifiedKFold* cross-validation scheme. AUROC values steadily increased when more synthetic samples were added, demonstrating that data augmentation improved classifier stability and discriminative capacity. The greatest performance gain came between 0 and 20 synthetic samples per group, after which improvements plateaued but remained directionally positive. With respect to this trend and the shrinking confidence intervals at higher augmentation levels, we determined that 20 synthetic samples (AUROC_mean ± AUROC_sd is 0.9638 ± 0.0412) per group provided the best balance of enhanced AUROC and modelling stability. Integrating SPsimSeq with LLM-generated profiles improves predictive performance in limited CD data sets.

**Figure 7:**
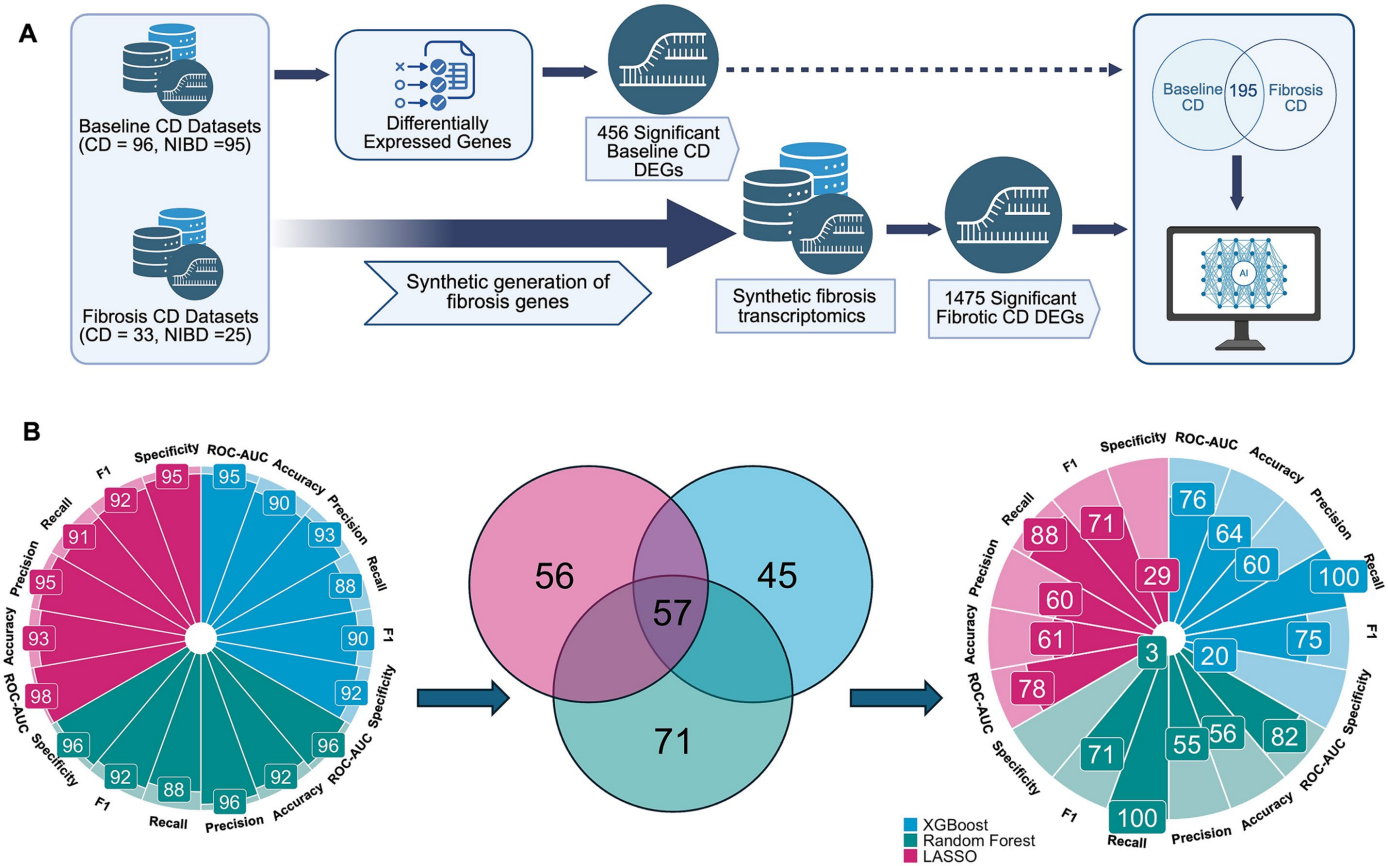
Large language model workflow and machine learning analysis. **(A)** The diagram illustrates the selection of 456 differentially expressed genes (DEGs) from the baseline CD dataset and the generation of fibrosis-related genes using large language model (LLM) approaches. A total of 1,475 genes were identified as significantly differentially expressed from the newly generated LLM dataset. The overlap between the baseline CD and fibrosis CD datasets resulted in 195 common genes, which were used as input features for machine learning models. **(B)** Circular bar plots display the performance metrics of the XGBoost, Random Forest, and LASSO models trained on the 195 common genes. This analysis identified 57 genes overlapping across all three models. These overlapping genes were further evaluated in the fibrosis dataset using the same machine learning models, and their performance scores were recorded.

Using the synthetic dataset, the machine learning pipeline was repeated. The intersection of DEGs between baseline CD and GPT-derived synthetic fibrosis genes identified 195 genes (Figure 7A). XGBoost, Random Forest, and LASSO models were retrained, and feature selection identified 102 genes for XGBoost, 128 for Random Forest, and 113 for LASSO. A total of 57 genes overlapped across all three models and were selected for predictive analysis. XGBoost achieved an AUC of 0.95 (0.93–0.98), Random Forest achieved 0.96 (0.94–0.98), and LASSO achieved 0.98 (0.96–1.00). The models were then evaluated on the synthetic fibrosis-oriented CD gene profiles to evaluate their ability to predict fibrosis-related alterations. Random Forest performed best, with an AUC of 0.82 (0.40–0.90), followed by LASSO 0.78 (0.49–0.79) and XGBoost 0.76 (0.54–0.77) (Figure 7B). Compared to models trained on the original dataset with fewer samples (AUC < 0.69), the models trained on the augmented dataset showed improved performance. This suggests that the synthetic fibrosis dataset reflects predefined biological patterns while introducing sufficient variability for effective classification (Supplementary Table 11).

In a second analysis, we evaluated the effect of incorporating an additional set of literature-derived fibrosis genes into the enhanced dataset, which included 20 synthetic samples per group. Using this expanded dataset, the Random Forest model showed strong discriminatory performance and the class distribution was balanced (Synthetic Fibrosis CD = 42 vs. NIBD = 35), and the model achieved an accuracy of 0.95 (0.85–1.00), ROC-AUC of 0.98 (0.93–1.00), precision of 1.00, recall of 0.88 (0.62–1.00), and an F1 score of 0.94 (0.76–1.00).

The confusion matrix showed only one misclassification, indicating consistent performance across groups. High cross-validation performance (CV AUC 0.92 ± 0.03) further suggests good generalisation across stratified folds. The synthetic fibrosis dataset identified key biomarkers, including IL-23R, TGF-*β*, and TNFα, consistent with their known roles in inflammation and fibrosis. Overall, these analyses indicate that synthetic augmentation combined with literature-informed gene expansion improves classification performance while preserving biologically relevant signals in CD.

### 3.11 Performance of the SPsimSeq–LLM framework

The global expression characteristics of the original data were preserved by both synthetic data generation approaches, as evidenced by the overall gene expression distributions being highly consistent across the original, SPsimSeq-generated, and LLM-generated datasets (Supplementary Figure 12A). The KL divergence distributions also showed similar patterns in all three datasets, indicating that the original gene expression profiles’ underlying distributional characteristics were preserved in the synthetic datasets (Supplementary Figure 12B). Additionally, Pearson correlation heatmaps showed that the SPsimSeq-generated dataset maintained the general correlation patterns, and the LLM-generated dataset nearly replicated the sample-to-sample correlation structure seen in the original dataset (Supplementary Figure 12C).

Random Forest classification performance was compared between the original, SPsimSeq-generated, and LLM-generated datasets to further assess the usefulness of the synthetic datasets for subsequent predictive analysis. Both synthetic datasets outperformed the original dataset in classification across several evaluation metrics, with the LLM-generated dataset doing best overall. In particular, the mean accuracy rose from (0.71) to (0.73) and (0.88), whereas the mean AUC improved from (Original: 0.79) to (SPsimSeq: 0.80) and (LLM: 0.94). The mean F1-score (0.73, 0.76, 0.88), etc., all showed comparable gains. Supplementary Table 12 provides a summary of the comparative classification results.

## 4 Discussion

Intestinal fibrosis is a complex and progressive complication of IBD, particularly CD. Although chronic inflammation initiates this process, fibrosis can become a self-sustaining, partially inflammation-independent condition that leads to tissue remodelling, strictures, and obstruction. The process is multifactorial and involves interactions between immune responses, microbial factors, and environmental influences.

In this study, we investigated transcriptomic and microbiome features associated with differences among control, inflammatory CD, and fibrotic CD using machine learning and statistical approaches. We also aimed to address the limited number of fibrosis transcriptomic samples and gaps in known fibrosis-associated genes by using a generative modelling approach to simulate fibrosis-related gene expressions.

### 4.1 Impact of transcriptomics factors in Crohn’s disease and intestinal fibrosis

was the identification of 94 genes shared between baseline CD and fibrosis CD when compared to NIBD with machine learning. Functional annotation revealed that these genes were enriched for immune and host-defence pathways, including inflammatory signalling, cytokine response, and neutrophil activation. The presence of a shared transcriptional core indicates that inflammatory and fibrosis CD are not distinct molecular states but instead share overlapping immune-associated transcriptional programs. This observation is consistent with previous work suggesting that intestinal fibrosis arises from persistent inflammatory signalling and dysregulated tissue repair processes rather than representing a completely independent molecular programme (Li and Kuemmerle, 2014; Rieder et al., 2016). To further refine these observations, machine learning was used to prioritise genes most strongly associated with differences across disease groups. Among the selected features, 27 genes exhibited significant differences among disease groups, highlighting distinct transcriptional patterns associated with inflammatory and fibrotic CD. A group of genes, including S100A8, TREM1, and CXCL1, primarily within Module 1, defined by correlation-based network clustering, were consistently downregulated in baseline CD but strongly upregulated in fibrosis CD. The genes are linked to innate immune activation and myeloid cell-mediated inflammatory responses. For instance, S100A8, a component of the calprotectin heterodimer (S100A8/S100A9), can bind to RAGE (Receptor for Advanced Glycation End Products) and TLR4 (Toll-like receptor 4) on intestinal fibroblasts, activating the NF-kB signalling. This may contribute to fibroblast proliferation and their transition into collagen-producing myofibroblasts, eventually leading to stricture formation in the colon (Touny et al., 2024; Fujita et al., 2018). Similarly, TREM1 is highly expressed on macrophages and neutrophils, and its upregulation is associated with fibroblast proliferation, and its inhibition has been shown to reduce immune activation (Schenk et al., 2007; Shi et al., 2025; Dharmasiri et al., 2021). Similarly, CXCL1 produced by activated fibroblasts contributes to fibrosis by promoting neutrophil recruitment to the intestinal mucosa. This supports a feedback loop in which immune cell infiltration sustains inflammation and drives fibrotic remodelling (Yang et al., 2024).

In contrast, the genes within Module 2, including FABP6, ALDOB, and MGAM, displayed the opposite pattern, being upregulated in baseline CD but downregulated in fibrosis. Collectively, these genes define a transcriptional programme associated with intestinal epithelial metabolic and absorptive function, as they encode proteins involved in bile acid transport and carbohydrate digestion. FABP6, expressed in ileal enterocytes, is essential for maintaining bile acid homeostasis, gut barrier integrity, and microbiota regulation. While FABP6 expression is significantly upregulated in active CD, it is profoundly depleted in fibrotic tissues. This loss of epithelial identity disrupts bile acid transport, reducing key bile acids contributing to dysbiosis and increased intestinal permeability (Heimerl et al., 2006; Kwon et al., 2025; Kim et al., 2023). Similarly, MGAM is a brush-border enzyme expressed in mature enterocytes that hydrolyses dietary carbohydrates to generate glucose. During inflammation, its upregulation may support epithelial repair by increasing energy availability (Sim et al., 2012; Park et al., 2021; Lee et al., 2012). As CD progresses to fibrosis, the loss of mature enterocytes leads to a decrease in MGAM, which reflects a decline in epithelial identity. Additionally, ALDOB catalyses the breakdown of fructose-1-phosphate into glycolytic intermediates that support ATP production (Tang and Cui, 2024). During intestinal inflammation, its upregulation may promote extracellular ATP release, activating P2X7 receptors and the NLRP3 inflammasome, leading to IL-1β and IL-18 production (Ülger and Delik, 2025; Santana et al., 2024). The coordinated suppression of these genes in fibrotic tissue samples suggests reduced epithelial metabolic capacity in fibrotic CD relative to inflammatory CD. A third group of genes within Module 3, including CHI3L1, SAA2-SAA4, and IL1RN, showed a heterogeneous pattern, consistent with cytokine-mediated inflammatory stress and epithelial immune signalling. Among these, CHI3L1 and IL1RN were more upregulated in fibrosis CD when compared to baseline CD.

CHI3L1 is a glycoprotein that promotes fibroblast and endothelial cell activity and is increased in fibrosis (Zhao et al., 2020). It is also involved in Akt and IL-6/STAT3 signalling, supporting a role in fibrotic remodelling. In addition, IL1RN, which encodes IL1Ra, regulates IL-1 signalling by inhibiting IL-1*α* and IL-1β receptor binding. Its increased expression in fibrosis CD likely reflects a compensatory response to persistent inflammation. As IL-1 signalling is also linked to extracellular matrix deposition, this upregulation might reflect an insufficient attempt to control fibrosis (Dosh et al., 2019; Ludwiczek et al., 2004; Zhang et al., 2025). Notably, IL1RN has also been identified as a biomarker in CRC, acting as a key modulator in the inflammatory tumour microenvironment (Burada et al., 2013). In contrast, SAA2-SAA4 was more upregulated in baseline CD. A study on paediatric IBD patients by (Siebert et al., 2025) reports that SAA2, from the SAA2-SAA4 complex, is involved in epithelial responses to microbial stimulation and promotes Th17 cell differentiation, thereby linking microbial sensing to persistent inflammation (Hazime et al., 2025; Grasberger et al., 2015).

In addition to the genes identified from transcriptomic analysis, two genes from GSVA analysis were also highlighted for their different roles in both baseline CD and fibrosis CD. Among these, LCN2 is strongly associated with inflammation severity and mucosal damage and is considered a sensitive biomarker of active CD. Its expression is regulated by the IL-23/Th17 axis, with IL-17A and TNF-α driving upregulation in intestinal epithelial cells and infiltrating neutrophils, reflecting active mucosal immune responses (Stallhofer et al., 2015). Beyond inflammation, LCN2 also contributes to intestinal fibrosis by promoting fibroblast activation and extracellular matrix production through TGF-β/SMAD and NF-κB signalling pathways, and its deletion reduces collagen deposition (Zhang et al., 2024b). In colorectal cancer, LCN2 exhibits context-dependent roles, with studies reporting both tumour-suppressive and pro-metastatic function (Dhami et al., 2026; Kim et al., 2017; Radhakrishnan et al., 2025).

Similarly, MMP3 is upregulated in inflamed intestinal tissue, especially in the submucosa and muscularis, promoting smooth muscle cell migration (Kofla-Dlubacz et al., 2011). In fibrosis CD, MMP3 contributes to extracellular matrix remodelling by degrading ECM components and activating other MMPs. However, its activity is often counterbalanced by increased levels of tissue inhibitors of metalloproteinases (TIMP-1), limiting effective ECM degradation. This imbalance favours collagen accumulation, contributing to intestinal thickening and stricturing. This is supported by GSVA results, which show that LCN2 and MMP3 change in their enrichment across disease stages, with LCN2 shifting across epithelial states and MMP3 becoming more associated with stromal populations in fibrosis CD.

### 4.2 Sensitivity analysis to address tissue heterogeneity in fibrosis NIBD cohort

In this study, XGBoost, Random Forest, and LASSO machine learning models trained on baseline CD transcriptomic profiles showed strong ability to distinguish CD from NIBD samples, with AUC values ranging from 0.95 (0.93–0.98) to 0.96 (0.94–0.98) across models. When the same models were applied to the fibrosis CD, performance decreased across all the classifiers with AUC values between 0.65 (0.49–0.79) and 0.69 (0.54–0.77). These findings indicate that although inflammatory and fibrotic CD share a common immune-associated transcriptional programme, the baseline inflammatory signature does not fully capture the molecular changes associated with fibrosis.

To determine whether these findings were influenced by the composition of the NIBD samples in the fibrosis cohort, a sensitivity analysis was performed by stratifying the controls into neoplastic, inflammatory, and non-inflammatory sub-groups. Model performance in the neoplastic and inflammatory groups was broadly comparable to that of the combined NIBD cohort, with slight improvements observed for XGBoost. This suggests that the overall predictive performance was not substantially affected by the heterogeneity of the NIBD controls. However, both the inflammatory (*n* = 5) and non-inflammatory (*n* = 4) subgroups included a limited number of control samples, resulting in less precise estimates of model performance, particularly for the non-inflammatory subgroup.

The reduced performance in fibrosis CD is likely driven by a combination of biological and other technical factors. Fibrosis represents a later stage of CD, characterised generally by tissue remodelling, fibroblast activation, and extracellular matrix deposition, alongside persistent inflammation (Mager et al., 2020). Our transcriptomic analyses identified a shared set of genes between baseline and fibrosis CD, and the network analysis further demonstrated that many of these genes remain highly coordinated across disease stages. In addition, the fibrosis cohort was considerably smaller than the baseline cohort, which may have introduced additional variability and reduced model generalisability.

### 4.3 Microbiome alterations associated with inflammatory and fibrotic transcriptional signatures

In addition to these transcriptomic changes, alterations in the gut microbiome were also observed across disease stages. The gut microbiome plays a critical role in maintaining intestinal homeostasis; disruption of the balanced microbial ecosystem, referred to as dysbiosis, has been widely implicated in the pathogenesis of gastrointestinal diseases, including CD.

In the microbiome analysis, several genera declined across disease groups, including *Faecalibacterium, Anaerostipes, Coprococcus,* and *Ruminococcus,* which are well-recognised producers of short-chain fatty acids (SCFAs), particularly butyrate, which plays a central role in maintaining epithelial barrier integrity and immune homeostasis.

This reduction in SCFA-producing genera may contribute to a shift toward a dysbiosis microbial state, which has been mathematically modelled as a transition from competitive to cross-feeding interactions within the microbiome (Corral López et al., 2026). Notably, some of these genera were also identified as part of a microbial signature derived from baseline CD using machine learning, which demonstrated robust discrimination from NIBD and retained predictive capacity for fibrotic CD. This persistence across disease stages suggests that key features of microbial dysbiosis are established early and may represent microbial features consistently associated with both inflammatory and fibrotic CD. This altered ecological structure promotes metabolic interdependence and sustained microbial signalling, potentially driving persistent activation of epithelial defence pathways observed in the transcriptomic data (Hsu et al., 2024).

As discussed previously, the depletion of FABP6 observed in fibrotic CD suggests impaired bile acid transport, potentially leading to increased luminal bile acid availability. This altered bile acid environment may favour the expansion of bile-tolerant taxa such as *Bilophila.* Members of this genus, particularly species such as *Bilophila wadsworthia*, are known to utilise taurine-conjugated bile acids and produce hydrogen sulphide (H₂S). Elevated H₂S production has been linked to epithelial barrier disruption and sustained mucosal inflammation, thereby contributing to a microenvironment that favours fibrotic remodelling (Sayavedra et al., 2025).

In addition, *Bacteroides* exhibited a clear trajectory in our analysis, showing significant enrichment as the disease progressed in fibrotic CD. Species within this genus are known to possess proteolytic activity and produce proteases capable of degrading components of the mucus layer and host proteins (Porras et al., 2022).

This may increase microbial contact with the intestinal epithelium, leading to barrier disturbance and local tissue stress (Desai et al., 2016). In response to such disruptions, the host may activate matrix metalloproteinases (MMPs), including MMP3, which was found to be upregulated in the transcriptomic analysis. As discussed previously, MMP3 is a key mediator of ECM degradation and tissue remodelling. While this process is initially part of a repair response, its persistent activation may become dysregulated, promoting excessive ECM turnover and contributing to fibrotic tissue formation (Ioannou et al., 2025).

Another upregulated gene identified in the transcriptomic analysis was LCN2. In addition to its role as an inflammation marker, LCN2 is a microbiota-inducible antimicrobial protein that restricts bacterial growth through iron sequestration. Its upregulation may therefore reflect increased microbial pressure and dysbiosis, aligning with the depletion of SCFA-producing taxa and the emergence of a more imbalanced intestinal environment in fibrotic disease (Singh et al., 2016). Previous studies have also linked specific microbial taxa to disease progression and fibrotic complications in CD. In particular, members of the *Ruminococcus* genus have been associated with stricturing phenotypes (Liu et al., 2025a).

### 4.4 Linking LLM-guided augmented fibrosis- signatures to microbiome driven inflammation

Building on these findings, we next examined how LLM-guided approaches could help capture fibrosis-associated signals that may be missed in conventional analyses. In this study, generative AI addressed several limitations of conventional RNA-seq workflows. The modelling framework would have been limited to the initial sample size and genes kept after conventional filtering in the absence of LLM-guided augmentation.

Due to low expression in subsets of samples, physiologically relevant genes, including those involved in interferon signalling and epithelial stress responses, were removed during filtering. The LLM-assisted approach enabled the inference of probable expression patterns for such genes, including IL-23R, TGF-*β*, and TNF*α*, from the broader transcriptomic structure, thereby preserving signals that would otherwise be lost.

The three genes identified are biologically relevant to intestinal fibrosis and reflect biological processes associated with inflammatory and fibrotic disease states. IL-23R signalling sustains Th17-driven immune activation, promoting the production of pro-inflammatory cytokines and maintaining chronic mucosal inflammation. Within this inflammatory environment, TNF-α further amplifies immune responses, driving epithelial damage and increasing intestinal permeability, which perpetuates tissue injury. Over time, this persistent inflammatory signalling creates conditions that favour activation of TGF-β pathways, which act as the primary drivers of fibrosis by promoting fibroblast differentiation, extracellular matrix deposition, and tissue remodelling (Theiss et al., 2005; Yun et al., 2019; Sisto and Lisi, 2023; Korta et al., 2023).

Additionally, these genes also have their implicated roles in the microbiome. For instance, certain species of *Ruminococcus* have been shown to produce inflammatory polysaccharides that activate TLR4-dependent signalling and induce pro-inflammatory cytokines such as TNF-α (Henke et al., 2019). In another instance, *Bacteroides* shows a context-dependent relationship with TGF-β signalling in intestinal inflammation and fibrosis. While certain species promote Treg responses and increase TGF-β and IL-10, supporting mucosal tolerance. In contrast, other species drive colitis when TGF-β signalling is impaired, but not when it is intact. This suggests that TGF-β restrains the pro-inflammatory effects of commensal bacteria (Ihara et al., 2017). On the other hand, *Faecalibacterium* is an anti-inflammatory bacterium that suppresses TNF-α signalling through butyrate production. Reduced levels of the microbe in CD are linked to increased TNF-α driven inflammation, contributing to tissue injury and fibrotic progression (Liu et al., 2025b). Additionally, in CRC, *Alistipes* is associated with increased TNF-α signalling, promoting chronic inflammation via TLR4 activation. This inflammatory state correlates with IL-6/LRG1 signalling and subsequent activation of TGF-β signalling, linking microbial-driven inflammation to tumour progression (Fu et al., 2024).

Our model was able to better capture transcriptome variability by adding k synthetic profiles to the original fibrosis RNA-Seq samples, increasing the sample size from n to n + k. By decreasing overfitting and increasing generalisation in ML models, this expansion directly raised AUC scores. To ensure that the dataset (Bulk RNA-Seq) expansion did not disrupt underlying biological structure, we first evaluated the range of synthetic samples and gene-level profiles that could be generated while maintaining global variance patterns. Based on this, an optimal augmentation size of 20 samples was selected, improving model stability while preserving coherence with the original cohort. Unlike conventional probabilistic simulators, which primarily increase sample size, our approach combines biologically constrained LLM prompting with SPsimSeq to enrich both samples and features. This feature-level enrichment is particularly relevant in IBD transcriptomics, where biologically important genes are often excluded due to sparsity and variability.

The findings show that the suggested SPsimSeq–LLM framework improves downstream prediction performance while maintaining physiologically significant gene expression patterns. Although SPsimSeq produces realistic RNA-seq distributions, it cannot recover low-count genes lost during preprocessing or reconstruct higher-order transcriptomic relationships. By integrating LLM-guided augmentation, our framework extends beyond sample generation to restore physiologically meaningful signals and stabilise model performance despite limited cohort size.

### 4.5 Current applications of generative AI in IBD research

While there are several ways to create synthetic datasets, RNA-seq-specific simulators like SPsimSeq provide a dependable means of creating gene-expression profiles that accurately reflect biological variation. SPsimSeq allowed for regulated dataset extension in our investigation while maintaining realistic count structures. Synthetic RNA-seq production is still underutilised in chronic inflammatory disorders like IBD, despite its widespread application in disciplines like immunology and oncology, where it could aid in fibrosis modelling, treatment-response prediction, and biomarker discovery. Simultaneously, biomedical modelling continues to change because of deep learning advancements. While platforms like PyEvoCell (Mathur et al., 2025) recreate cellular pathways and contextualise differential expression results, EHR-based solutions now allow enhanced natural language interpretation, patient stratification, and flare prediction. Similarly, LLMs used to EHR data have demonstrated how generative and language-based models can recover latent structure in sparse or incomplete datasets through advanced predictive modelling and risk profiling (Park et al., 2025).

Recent work in IBD informatics has demonstrated an increasing interest in applying LLMs to unstructured clinical data, going beyond tools like SPsimSeq and PyEvoCell. Research assessing GPT-4, PaLM-2, and IBDBERT shows good results in identifying serious adverse events, extracting PROs, and categorising CD from imaging reports. These results frequently surpass rule-based or conventional NLP techniques while still being highly generalisable across institutions (Patel et al., 2024; Dai et al., 2025). Further analyses of ChatGPT for demographic fairness, patient education, and compliance with ECCO criteria show both the potential and instability of existing models, such as drift, hallucinations, and biased suggestions (Omar et al., 2025; Ozturk et al., 2025; Yan et al., 2025).

These developments highlight the benefits of LLM-enabled harmonisation across various clinical literature, but they also highlight shortcomings that our method tackles by concentrating on physiologically grounded feature-level augmentation of RNA-seq data aspect where LLM use cases for IBD are still few.

### 4.6 Clinical implications

Although this study is based on retrospective public datasets and does not include prospective clinical validation, the findings suggest several potential directions for future translational research. One of the major unmet challenges in clinical practice is distinguishing active inflammation from established fibrosis, as these processes frequently coexist and cannot always be reliably differentiated using current endoscopic or cross-sectional imaging techniques alone (Rieder et al., 2024). In this context, the stage-associated molecular signatures identified here may provide complementary molecular information by capturing biological processes associated with fibrostenotic disease, including immune activation, extracellular matrix remodelling, and epithelial dysfunction. If validated prospectively, these signatures could contribute to future strategies for identifying patients at increased risk of fibrostenotic complications, supporting closer surveillance, earlier imaging assessment, or enrolment into clinical trials evaluating emerging antifibrotic therapies. Furthermore, the concurrent identification of host transcriptomic and microbiome alterations suggests that integrated molecular profiling may provide greater biological insight than either modality alone and could inform the development of multimodal biomarker panels combining molecular, microbial, imaging, and clinical data. Finally, the machine learning and LLM-guided framework presented here provides a reproducible approach for prioritising candidate biomarkers from small, heterogeneous transcriptomic datasets, supporting their subsequent evaluation in independent cohorts and prospective studies. However, these potential applications require validation in prospective clinical studies before implementation in routine clinical practice (Agweyu et al., 2026; Russ et al., 2026).

## 5 Limitations

Several factors limit this study. First, the lack of longitudinal datasets limits the ability to track transcriptomic and microbiome changes over time, making it difficult to fully characterise the progression from inflammation to intestinal fibrosis. Second, incomplete and inconsistent metadata across datasets, including BMI, medication use, disease duration, race, and dietary factors, may introduce bias and confound the interpretation of the results. In addition, the datasets were derived from multiple studies with different sequencing platforms and protocols. Although batch correction methods such as ComBat were applied, residual technical variation may still influence the findings. There were also imbalances in sample sizes used for machine learning, with fewer fibrosis samples than baseline CD samples, which may have affected model performance and reduced predictive accuracy. Furthermore, microbiome analysis was performed at the genus level, potentially overlooking species- or strain-specific effects relevant to fibrogenesis. The identified genes and microbial features were not validated in independent cohorts, limiting the generalisability of the results.

Finally, while LLM-guided modelling improved feature retention and dataset expansion, it also introduces additional limitations. The synthetic augmentation approach relies on inferred expression patterns rather than true biological measurements and, therefore, may not fully capture complex transcriptomic variability.

In addition, the models could not account for missing or poorly annotated metadata, and the integration of heterogeneous datasets may introduce further bias despite correction. External validation in independent CD cohorts will be necessary to determine the robustness and generalisability of the LLM-guided framework. Together, these limitations highlight the need for larger, well-phenotyped, and longitudinal datasets to confirm these findings.

## 6 Conclusion

In conclusion, this study uses an integrated approach combining transcriptomics, microbiome analysis, and generative AI to characterise molecular features associated with inflammatory and fibrotic CD. Machine learning and pathway analysis identified core transcriptomic modules associated with inflammation and fibrosis, highlighting that fibrotic CD retains core inflammatory features while acquiring additional epithelial and metabolic alterations. These transcriptomic changes were accompanied by shifts in the gut microbiome, particularly the loss of SCFA-producing genera and the enrichment of microbes linked to inflammatory signalling, supporting a role for host-microbiome interactions associated with fibrotic disease. By addressing limitations in sample size and feature sparsity, the use of LLM-guided approaches improved model stability and facilitated the identification of biologically relevant genes, including IL-23R, TNF-*α*, and TGF-*β*. When considered collectively, these findings suggest that intestinal fibrosis is unlikely to represent a distinct endpoint, but rather reflects a reconfigured inflammatory state driven by ongoing immune activation, loss of epithelial integrity, and interactions between the host and the microbiome.

## Data Availability

This study analysed publicly available transcriptomic and microbiome datasets obtained from the GEO, the Single Cell Portal, and NCBI BioProject. The datasets used in this study are GSE230113, GSE139179, GSE83687, GSE266546, GSE123141, GSE66207, GSE67250, SCP2959, and PRJNA398187. Direct access to these datasets is provided through their respective accession records. The analysed datasets have been deposited in Figshare and can be accessed via this link: https://doi.org/10.6084/m9.figshare.31946523. The Python and R packages used in this study can be found in Supplementary Table 1, and the underlying code generated for this study has been deposited in Figshare and can be accessed via this link: https://doi.org/10.6084/m9.figshare.31946598.
