# Supplementary material for: Generative AI-augmented transcriptomic and microbiome analysis across inflammatory and fibrotic disease states in Crohn’s disease
Source: Front Artif Intell. 2026 Aug 4;9:1881820. doi: 10.3389/frai.2026.1881820 (PMC13484702; doi:10.3389/frai.2026.1881820)
Supplement: Supplementary file 1 [file Supplementary_file_1.docx]

**Generative AI-Augmented Transcriptomic and Microbiome Analysis Across Inflammatory and Fibrotic Disease States in Crohn's Disease**

Daryll Philip^1†^, Daniela Santos^1†^, Sudip Mondal^2,3,4†^, Haneen Alomar^2,5^,

Georgios Gkoutos^2,3,4^, Animesh Acharjee^1,2,3,4*^

1. Cancer and Genomic Sciences, School of Medical Sciences, College of Medicine and Health, University of Birmingham Dubai, Dubai, UAE

2. Cancer and Genomic Sciences, School of Medical Sciences, College of Medicine and Health, University of Birmingham, Edgbaston, UK

3. Centre for Health Data Research, University of Birmingham, Birmingham, B15 2TT, UK

4. Institute of Translational Medicine, University Hospitals Birmingham NHS, Foundation Trust, UK

5. Department of Research Laboratories, King Faisal Specialist Hospital & Research Centre, Jeddah, Saudi Arabia

† Equal contribution: These authors contributed equally to this work

***Correspondence:**

Dr. Animesh Acharjee

Cancer and Genomic Sciences

University of Birmingham, B15 2TT, Birmingham, UK

E-mail: [a.acharjee@bham.ac.uk](mailto:a.acharjee@bham.ac.uk)

**SUPPLEMENTARY METHODS**

**Supplementary Method 1: Data Acquisition**

Data acquisition followed a structured, PRISMA-guided strategy(Page et al., 2021) to systematically identify and curate transcriptomic and microbiome datasets relevant to Crohn’s disease (CD) progression and fibrosis. Public repositories were queried, primarily the Gene Expression Omnibus (GEO)(Clough and Barrett, 2016), alongside complementary sources including PubMed and OmicsDI (Perez-Riverol et al., 2017), using predefined keyword combinations encompassing “Crohn’s disease,” “IBD,” “RNA-seq,” “scRNA-seq,” “transcriptomics,” “fibrosis,” “biopsy,” and “colon.” Initial screening yielded a broad pool of candidate studies, which were subsequently filtered through title, abstract, and full-text review according to predefined inclusion criteria: (i) human adult samples, (ii) intestinal biopsy-derived tissue (colon and/or ileum), (iii) availability of bulk or single-cell transcriptomic data, and (iv) clearly defined clinical phenotypes, including non-IBD controls, baseline CD, and fibrotic CD or treatment-response categories. Studies were excluded if they focused on paediatric populations, non-intestinal tissues, organoid or in vitro systems, non-transcriptomic modalities (unless complementary, e.g., microbiome 16S), lacked accessible raw or processed data, or had insufficient clinical annotation. After systematic screening, a final set of multi-cohort datasets was retained, including RNA-seq and scRNA-seq studies, as well as one microbiome dataset to support host–microbe interaction analyses. For each dataset, raw count matrices or normalized expression data, along with corresponding metadata (e.g., age, sex, biopsy location, disease status, fibrosis classification, and treatment information), were downloaded from GEO series matrix files or associated supplementary files (e.g., family XML). Samples were further curated to remove duplicates, resolve inconsistencies in clinical annotation, and ensure comparability across cohorts. Importantly, datasets were initially processed independently to preserve platform-specific characteristics before downstream normalisation and integration. This rigorous acquisition and selection framework ensured the inclusion of high-quality, well-annotated, and biologically relevant datasets, enabling robust cross-cohort analyses of transcriptional changes across the continuum from non-IBD tissue to inflammatory and fibrotic Crohn’s disease.

**Supplementary Method 2: Cell-Type Signature Scoring and Deconvolution**

The scRNA-seq datasets from baseline CD and fibrosis CD patients were preprocessed individually before downstream analyses. The scRNA-seq dataset from baseline CD patients was processed to retain high-quality cells. Cells with low transcript complexity or poor quality were filtered based on the number of detected genes, total UMI counts, and mitochondrial gene expression. Specifically, cells with fewer than 200 detected genes, more than 6000 detected genes, or mitochondrial transcript content ≥20% were excluded to remove potential doublets. Genes expressed in only a small number of cells were also removed.

Following quality control, gene expression counts were normalised using log-normalization, and highly variable genes were identified. Expression values were scaled while regressing out technical effects such as total UMI counts and mitochondrial gene content. Dimensionality reduction was performed using PCA on the highly variable genes, followed by graph-based clustering and visualisation using Uniform Manifold Approximation and Projection (UMAP) (Mcinnes et al., 2020). Cell-type identities were assigned based on canonical marker gene expression.

For the fibrosis CD scRNA-seq datasets, differentially expressed genes identified from bulk RNA-seq analysis were examined within the single-cell data. To retain high-quality cells, filtering thresholds were applied based on quality control metrics and visual inspection of QC plots. Lower thresholds were used to remove empty droplets or cells with minimal gene activity, retaining only cells with at least 10 detected genes. Upper thresholds were applied to remove potential doublets displaying unusually high RNA content; specifically, cells with more than 65 detected genes or more than 55 total counts were excluded. Cells with mitochondrial transcript proportions greater than 20% were also removed. Cells were normalised by log-transformation, and marker genes for each cell cluster were identified using the Wilcoxon rank-sum test. The top 200 marker genes per cluster were selected to construct cell-type-specific gene sets. The filtered dataset was retained for downstream analysis and integration with bulk transcriptomic profiles.

**Supplementary Method 3**: **Uniform Manifold Approximation and Projection (UMAP) and Feature Plot Generation.**

To visualise the cellular localisation of genes identified from the baseline CD and fibrosis CD bulk RNA-seq analyses, representative genes from each co-expression module were mapped onto the corresponding baseline CD and fibrosis CD single-cell RNA-seq datasets, respectively. The scRNA-seq data were processed and analysed using the Seurat package. Cells were normalised, clustered, annotated, and visualised using the FeaturePlot () function, which projects normalised gene expression onto the UMAP embedding. Individual feature plots were generated for each selected gene, with cells coloured according to normalised expression intensity to illustrate their distribution across annotated cell populations.

**Supplementary Method 4: Synthetic Data Generation and Modelling Framework (Prompt Engineering)**

In our analysis, fixed prompt templates were used for both the CD and NIBD profiles to ensure repeatability. The CD prompt included fibrosis severity categories ("mild," "moderate," "severe") and inflammatory states ("low," "mixed," "high"), which corresponded to projected gene-level regulation. The NIBD template simulated expression ranges in the absence of inflammation or fibrosis. Both prompts limited output to basic gene-value combinations within the acceptable CPM limits. The samples of the prompt structure utilised in this work are shown below:

**Healthy Control Prompt:**

" ... generating synthetic RNA-seq gene expression profiles (CPM scale).

Condition: Healthy control (no inflammation, no fibrosis)..

Genes of interest:

<same gene list>

Rules:

- Output only gene-value pairs

- Format: GENE, VALUE

- VALUE must be a float

- Range: 0–5000 CPM... “

**Fibrosis Crohn’s Disease Prompt:**

“ ... generating synthetic RNA-seq gene expression profiles (CPM scale).

Condition: Crohn's Disease with intestinal fibrosis...

Genes of interest:

<list of fibrosis-associated genes>

Biological constraints:

- Fibrosis genes are mildly, moderately, or strongly upregulated depending on severity

- Immune and stromal expression adjusted according to inflammation level

- Differentially expressed genes must follow disease direction

Rules:

- Output only gene-value pairs

- Format: GENE,VALUE

- VALUE must be a float

- Range: 0–5000 CPM ... ”

**SUPPLEMENTARY TABLES**

**Supplementary Table 1: Computational and statistical packages.** Summary of the R and Python packages utilised in this study.

| **Tools/Packages** | **Analysis Step** | **References** |
| --- | --- | --- |
| **R (v4.3.2)** | Statistical computing | https://www.r-project.org/ |
| **DESeq2** | Bulk RNA-seq normalisation and DGE analysis | https://bioconductor.org/packages/DESeq2/ |
| **Sva(ComBat)** | Batch Correction | https://bioconductor.org/packages/sva/ |
| **Seurat** | Sc-RNA-seq clustering | <https://satijalab.org/seurat/> |
| **apeglm** | Log-fold change | https://bioconductor.org/packages/apeglm/ |
| **edgeR** | Differential expression framework | https://bioconductor.org/packages/edgeR/ |
| **limma** | Linear modelling for RNA-seq / microarray | https://bioconductor.org/packages/limma/ |
| **biomaRt** | Gene annotation and ID mapping | https://bioconductor.org/packages/biomaRt/ |
| **org.Hs.eg.db** | Gene annotation database | https://bioconductor.org/packages/org.Hs.eg.db/ |
| **msigdbr** | Pathway gene set retrieval | https://cran.r-project.org/package=msigdbr |
| **EnhancedVolcano** | Differential expression visualisation | https://bioconductor.org/packages/EnhancedVolcano/ |
| **matrixStats** | Vectorised statistics | https://cran.r-project.org/package=matrixStats |
| **vegan** | Alpha and Beta Diversity | https://github.com/vegandevs/vegan |
| **ape** | PCoA from the Bray–Curtis distance matrix | https://cran.r-project.org/web/packages/ape/index.html |
| **FSA** | Post hoc testing | https://cran.r-project.org/web/packages/FSA/index.html |
| **pheatmap** | To generate heatmaps | https://cran.r-project.org/web/packages/pheatmap/index.html |
| **Python (v3.9.6)** | Machine Learning & LLM | https://www.python.org/ |
| **StratifiedKFold** | Stratified cross-validation | https://scikit-learn.org/stable/modules/generated/sklearn.model_selection.StratifiedKFold.html |
| **GridSearchCV** | Hyperparameter tuning | https://scikit-learn.org/stable/modules/generated/sklearn.model_selection.GridSearchCV.html |
| **scikit-learn** | Machine learning (Random Forest & LASSO) | https://github.com/scikit-learn/scikit-learn |
| **Scanpy** | Single cell preprocessing | https://github.com/scverse/scanpy |
| **pandas** | Data handling & preprocessing | https://github.com/pandas-dev/pandas |
| **NumPy** | Numerical computations | https://github.com/numpy/numpy |
| **BorutaPy** | Feature selection | https://github.com/scikit-learn-contrib/boruta_py |
| **seaborn** | Data visualisation | https://github.com/mwaskom/seaborn |
| **optuna** | Hyperparameter tuning | https://github.com/optuna/optuna |
| **xgboost** | Machine learning (XGBoost) | https://github.com/dmlc/xgboost |
| **matplotlib.pyplot** | Data visualisation | https://github.com/matplotlib/matplotlib |
| **GPT-4.1 mini** | OpenAI API | https://developers.openai.com/api/docs/models/gpt-4.1-mini |
| **SPsimSeq** | Simulation of bulk RNA-seq data | https://github.com/CenterForStatistics-UGent/SPsimSeq |

**Supplementary Table 2. Variance Filtering for Baseline CD and Fibrosis CD Datasets.** The table represents the number of genes before variance and after variance filtering

| **Dataset Type** | **Dataset** | **Genes before variance filter** | **Genes removed** | **Genes after variance filter** | **Variance cutoff** | **% of genes retained** |
| --- | --- | --- | --- | --- | --- | --- |
| Baseline CD | GSE230113 | 36,155 | 6,911 | 27,630 | 2.326316 | 80% |
|  | GSE83687 | 38,815 | 7,421 | 29,684 | 1.267871 | 80% |
|  | GSE139179 | 36,312 | 6,938 | 27,752 | 0.317214 | 80% |
| Fibrosis CD | GSE123141 | 39,376 | 16,870 | 22,506 | 0.596629 | 80% |
|  | GSE66207 | 39,376 | 8,697 | 30,679 | 0.251686 | 80% |
|  | GSE67250 | 39,376 | 16,231 | 23,145 | 0.109989 | 80% |

**Supplementary Table 3. Performance metrics for transcriptomics.** Scores were calculated along with their 95% confidence intervals for Baseline CD and Fibrosis CD bulk-RNA transcriptomics data for XGBoost, Random Forest, and LASSO models.

| **Data** | **Classifier** | **AUC(%)** | **Accuracy(%)** | **Precision(%)** | **Recall(%)** | **F1(%)** | **Specificity(%)** |
| --- | --- | --- | --- | --- | --- | --- | --- |
| **Training**  Baseline CD | XGBoost | 0.95  (0.93-0.98) | 0.87  (0.82-0.93) | 0.89  (0.81-0.97) | 0.88  (0.84-0.92) | 0.88  (0.83-0.92) | 0.87  (0.78-0.97) |
|  | Random Forest | 0.96  (0.94-0.98) | 0.90  (0.86-0.93) | 0.94  (0.89-0.98) | 0.86  (0.80-0.92) | 0.90  (0.86-0.94) | 0.94  (0.89-0.98) |
|  | LASSO | 0.96  (0.94-0.98) | 0.92  (0.89-0.96) | 0.93  (0.90-0.97) | 0.92  (0.89-0.96) | 0.92  (0.88-0.96) | 0.93  (0.87-0.99) |
| **Prediction**  Fibrosis  CD | XGBoost | 0.69  (0.54-0.77) | 0.68  (0.54-0.81) | 0.67  (0.51-0.80) | 0.88  (0.74-0.97) | 0.76  (0.62-0.85) | 0.44  (0.26-0.64) |
|  | Random Forest | 0.65  (0.49-0.79) | 0.60  (0.47-0.72) | 0.63  (0.46-0.77) | 0.69  (0.53-0.84) | 0.66  (0.52-0.77) | 0.48  (0.27-0.65) |
|  | LASSO | 0.65  (0.49-0.79) | 0.60  (0.47-0.72) | 0.62  (0.46-0.76) | 0.72  (0.57-0.88) | 0.67  (0.53-0.78) | 0.44  (0.24-0.64) |

**Supplementary Table 4: Best performing hyperparameters for the transcriptomics machine learning models.** These are the best hyperparameters that are used for training the baseline transcriptomics models, subsequently used for the microbiome prediction models.

| **Machine Learning Model** | **Hyperparameters** | **Value** |
| --- | --- | --- |
| **XGBoost** | n_estimators | 115 |
|  | booster | gbtree |
|  | learning_rate | 0.202841 |
|  | gamma | 0.455652 |
|  | max_depth | 3 |
|  | min_child_weight | 4.344663 |
|  | subsample | 0.544676 |
|  | colsample_bytree | 0.870110 |
|  | reg_lambda | 2.424727e-01 |
|  | alpha | 3.920680e-04 |
|  | random_state | 42 |
|  |  |  |
| **Random Forest** | n_estimators | 51 |
|  | max_depth | 11 |
|  | min_samples_split | 2 |
|  | min_samples_leaf | 2 |
|  | max_features | sqrt |
|  | random_state | 42 |
|  |  |  |
| **LASSO** | penalty | L1 |
|  | C | 0.152971 |
|  | solver | saga |
|  | max_iter | 10000 |
|  | random_state | 42 |

**Supplementary Table 5: Prediction performance metrics for different non-IBD (NIBD) subgroups.** As a part of the sensitivity analysis, the NIBD controls in the fibrosis cohort were divided into three biologically relevant subgroups. This table presents the prediction results for fibrosis CD using neoplastic (adenoma, polyps, and neuroendocrine tumour), inflammatory (diverticulitis), and non-inflammatory (colonic inertia and iron deficiency anaemia) conditions, respectively.

| **Data** | **Classifier** | **AUC** | **Accuracy** | **Precision** | **Recall** | **F1** | **Specificity** |
| --- | --- | --- | --- | --- | --- | --- | --- |
| **Prediction**  Fibrosis  CD: 32  Neoplastic NIBD: 12 | XGBoost | 0.75  (0.53-0.83) | 0.77  (0.53-0.83) | 0.76  (0.69-0.94) | 0.77  (0.53-0.83) | 0.77  (0.53-0.83) | 0.50  (0.21-0.78) |
|  | Random Forest | 0.66  (0.49-0.83) | 0.61  (0.45-0.75) | 0.76  (0.59-0.90) | 0.69  (0.53-0.85) | 0.72  (0.58-0.84) | 0.42  (0.11-0.72) |
|  | LASSO | 0.69  (0.52-0.85) | 0.64  (0.50-0.77) | 0.77  (0.61-0.91) | 0.72  (0.56-0.87) | 0.74  (0.61-0.85) | 0.42  (0.14-0.71) |
| **Prediction**  Fibrosis  CD: 32  Inflammatory NIBD: 5 | XGBoost | 0.68  (0.41-0.93) | 0.81  (0.68-0.92) | 0.83  (0.79-1.00) | 0.81  (0.75-0.97) | 0.82  (0.79-0.96) | 0.40  (0.27-0.65) |
|  | Random Forest | 0.62  (0.38-0.81) | 0.65  (0.49-0.81) | 0.88  (0.74-1.00) | 0.69  (0.52-0.84) | 0.77  (0.64-0.89) | 0.40  (0.27-0.65) |
|  | LASSO | 0.72  (0.41-0.95) | 0.70  (0.57-0.86) | 0.92  (0.79-1.00) | 0.72  (0.56-0.87) | 0.81  (0.68-0.91) | 0.60  (0.49-0.87) |
| **Prediction**  Fibrosis  CD: 32  Non-Inflammatory NIBD: 4 | XGBoost | 0.45  (0.39-0.92) | 0.81  (0.67-0.92) | 0.83  (0.79-1.00) | 0.81  (0.76-0.97) | 0.81  (0.80-0.96) | 0.25  (0.12-0.35) |
|  | Random Forest | 0.47  (0.08-0.89) | 0.67  (0.50-0.81) | 0.92  (0.79-1.00) | 0.69  (0.51-0.84) | 0.79  (0.64-0.89) | 0.50  (0.27-0.65) |
|  | LASSO | 0.39  (0.07-0.75) | 0.67  (0.50-0.81) | 0.88  (0.75-1.00) | 0.72  (0.56-0.87) | 0.79  (0.67-0.89) | 0.25  (0.12-0.35) |

**Supplementary Table 6: Log2 fold change results.** 27 genes showed a significant log-fold change moving from NIBD to Baseline CD to Fibrosis CD. Positive values indicate upregulation, whereas negative values indicate downregulation.

| **Gene** | **Log2FC (CD)** | **Log2FC (Fibrosis)** |
| --- | --- | --- |
| **PROK2** | -0.311 | 5.659 |
| **CHI3L1** | -0.047 | 4.843 |
| **FABP6** | 8.502 | -1.241 |
| **MGAM** | 6.97 | -0.555 |
| **ALDOB** | 5.919 | -1.549 |
| **TREM1** | -1.315 | 3.861 |
| **KCNJ15** | -0.651 | 3.681 |
| **FCGR1BP** | -0.769 | 2.723 |
| **CXCL1** | -1.765 | 3.209 |
| **SLC7A9** | 5.188 | -0.832 |
| **CLEC4E** | -1.998 | 2.565 |
| **S100A8** | -0.896 | 3.015 |
| **IL1RN** | 0.481 | 1.91 |
| **CXCR1** | 0.503 | 2.51 |
| **C17orf78** | 4.91 | -0.096 |
| **CRABP2** | -2.022 | 1.199 |
| **FCGR1CP** | -0.776 | 1.903 |
| **ANPEP** | 2.624 | -1.92 |
| **ELK2AP** | 2.011 | 0.133 |
| **HCAR3** | 0.176 | 2.241 |
| **NLRP6** | 2.654 | -0.558 |
| **SAA2-SAA4** | 1.742 | 0.164 |
| **DUOXA2** | 1.838 | -1.343 |
| **RNA5-8SN3** | -1.557 | 2.094 |
| **SMLR1** | 3.36 | -0.334 |
| **ACE** | 1.572 | 0.156 |
| **HSPB3** | 0.995 | -3.091 |

**Supplementary Table 7: Hub genes and module assignment from the co-expression network.** Genes identified from the correlation network are grouped by module and ranked by strength (node connectivity). Strength is the sum of edge weights (connectivity); higher values indicate more central (hub-like) genes.

| **Gene** | **Module** | **Strength** |
| --- | --- | --- |
| **S100A8** | 1 | 7.833479042884990 |
| **TREM1** | 1 | 7.260825322427710 |
| **HCAR3** | 1 | 7.060301256354200 |
| **CXCR1** | 1 | 7.037190074528260 |
| **CHI3L1** | 2 | 7.021288440887340 |
| **CLEC4E** | 1 | 6.8700037273707800 |
| **CXCL1** | 1 | 5.436058025808120 |
| **KCNJ15** | 1 | 5.0126873639421700 |
| **PROK2** | 1 | 4.801715122775580 |
| **FCGR1BP** | 1 | 4.759189605974850 |
| **FCGR1CP** | 1 | 4.612251415602460 |
| **ALDOB** | 3 | 2.0335265065365400 |
| **FABP6** | 3 | 1.398560337830380 |
| **SAA2-SAA4** | 2 | 1.3614495260794000 |
| **DUOXA2** | 2 | 1.3485245482326100 |
| **IL1RN** | 2 | 1.2062175578802800 |
| **MGAM** | 3 | 0.6656785164056870 |
| **SLC7A9** | 3 | 0.6385127539739680 |
| **ANPEP** | 3 | 0.6078004062744380 |

**Supplementary Table 8: Significant cell types in baseline CD and fibrosis CD from GSVA analysis.** The table summarises the 11 significant cell types in baseline CD (FDR < 0.05) and 26 in fibrosis CD (FDR < 0.01) associated with genes derived from the 43-gene machine learning signature. Genes with significant GSVA associations are presented.

| **Genes** | **Cell types in Baseline CD** | **Cell types in Fibrosis CD** |
| --- | --- | --- |
| **ALDOB** | LND; EEC | Enterocytes (COX-hi) |
| **DUOX2** | Goblet Proliferating; Early Goblet; LND | Enterocytes (mito-hi) |
| **IGHG2** | Plasma; Endo.; Recruited Macro.; Neutrophils | Regulatory T cells; Dendritic cells (cDC1); Monocytes (CCL3/4-hi); Arterial endothelium; Cycling myeloid; GZMK-hi CD8 T cells; Inflammatory fibroblasts; IgA plasma cells; Mito-hi endothelium |
| **IGHG4** | Plasma; Fibro.; Neutrophils | Dendritic cells (CCR7-hi); Dendritic cells (cDC1); Monocytes (CCL3/4-hi); Cycling myeloid; M2-like macrophages (A2M-hi); Monocytes; Monocytes (HSP-hi); Inflammatory fibroblasts; IgA plasma cells |
| **LCN2** | Goblet Proliferating; Early Goblet; LND | Colonocytes (LGALS4-hi); Stem-like cells (OLFM4-hi); Enterocytes (mito-hi) |
| **MMP3** | Fibro. | Telocytes, Inflammatory fibroblasts |
| **NOS2** | Goblet Proliferating; LND | Stem-like cells (OLFM4-hi), Enterocytes (mito-hi), Enterocytes (COX-hi) |
| **S100A8** | Neutrophils | CD63+CD81+ macrophages; Dendritic cells (cDC2); Monocytes (CCL3/4-hi); Tissue macrophages; Monocytes (FPR1-hi); Tissue macrophages (KRT-hi); Monocytes; Monocytes (HSP-hi); NAMPT-hi neutrophils; Mito-hi endothelium |
| **S100A9** | Neutrophils | Dendritic cells (CCR7-hi); CD63+CD81+ macrophages; Dendritic cells (cDC2); Monocytes (CCL3/4-hi); M2-like macrophages; Tissue macrophages; Cycling myeloid; Monocytes (FPR1-hi); Tissue macrophages (KRT-hi); Monocytes; Monocytes (HSP-hi); NAMPT-hi neutrophils; Mito-hi endothelium |

| **Data** | **Classifier** | **AUC(%)** | **Accuracy(%)** | **Precision(%)** | **Recall(%)** | **F1(%)** | **Specificity**  **(%)** |
| --- | --- | --- | --- | --- | --- | --- | --- |
| **Training**  Baseline CD  (Microbiome) | XGBoost | 0.69  (0.50-0.88) | 0.62  (0.45-0.77) | 0.69  (0.44-0.93) | 0.68  (0.56-0.81) | 0.67  (0.56-0.78) | 0.57  (0.19-0.96) |
|  | Random Forest | 0.74  (0.56-0.92) | 0.68  (0.58-0.78) | 0.744  (0.61-0.87) | 0.62  (0.47-0.78) | 0.67  (0.54-0.82) | 0.75  (0.65-0.84) |
|  | LASSO | 0.80  (0.67-0.94) | 0.71  (0.57-0.85) | 0.71  (0.58-0.85) | 0.75  (0.59-0.91) | 0.73  (0.59-0.87) | 0.66  (0.58-0.81) |
| **Prediction**  Fibrosis  CD  (Microbiome) | XGBoost | 0.81  (0.50-1.00) | 0.83  (0.70-0.96) | 0.60  (0.08-0.99) | 0.36  (0.14-0.88) | 0.43  (0.10-0.97) | 1.00  (0.99-1.00) |
|  | Random Forest | 0.82  (0.58-1.00) | 0.85  (0.73-0.98) | 0.80  (0.24-0.99) | 0.46  (0.01-0.91) | 0.56  (0.11-0.99) | 1.00  (0.99-1.00) |
|  | LASSO | 0.84  (0.64-1.00) | 0.72  (0.59-0.84) | 0.36  (0.08-0.64) | 0.60  (0.23-0.96) | 0.43  (0.14-0.72) | 0.75  (0.58-0.92) |

**Supplementary Table 9: Performance metrics for microbiome.** Scores were calculated along with their 95% confidence intervals for Baseline CD and Fibrosis CD microbiome datasets for XGBoost, Random Forest, and LASSO models.

| **Machine Learning Model** | **Hyperparameters** | **Value** |
| --- | --- | --- |
| **XGBoost** | n_estimators | 400 |
|  | learning_rate | 0.1 |
|  | gamma | 0.455652 |
|  | max_depth | 3 |
|  | subsample | 0.7 |
|  | colsample_bytree | 1.0 |
|  | reg_lambda | 1 |
|  | reg_alpha | 1 |
|  | random_state | 42 |
|  |  |  |
| **Random Forest** | n_estimators | 400 |
|  | max_depth | None |
|  | min_samples_split | 10 |
|  | min_samples_leaf | 1 |
|  | random_state | 42 |
|  |  |  |
| **LASSO** | C | 0.885867 |

**Supplementary Table 10: Best performing hyperparameters for the microbiome machine learning models.** These are the best hyperparameters that are used for training the baseline transcriptomics models, subsequently used for the microbiome prediction models.

**Supplementary Table 11: Performance metrics.** Scores were calculated along with their 95% confidence intervals for the Baseline CD bulk RNA-seq and Fibrosis CD LLM-generated bulk RNA-seq dataset for XGBoost, Random Forest, and LASSO models.

| **Data** | **Classifier** | **AUC(%)** | **Accuracy(%)** | **Precision(%)** | **Recall(%)** | **F1(%)** | **Specificity(%)** |
| --- | --- | --- | --- | --- | --- | --- | --- |
| **Training**  Baseline CD | XGBoost | 0.95  (0.93-0.98) | 0.90  (0.87-0.94) | 0.93  (0.89-0.97) | 0.88  (0.84-0.93) | 0.90  (0.87-0.93) | 0.92  (0.84-0.93) |
|  | Random Forest | 0.96  (0.94-0.98) | 0.92  (0.90-0.95) | 0.96  (0.92-1.00) | 0.88  (0.84-0.93) | 0.92  (0.89-0.94) | 0.96  (0.91-1.00) |
|  | LASSO | 0.98  (0.96-1.00) | 0.93  (0.89-0.96) | 0.95  (0.90-0.99) | 0.91  (0.86-0.95) | 0.92  (0.89-0.96) | 0.95  (0.91-0.99) |
| **Prediction**  Fibrosis  CD  (LLM) | XGBoost | 0.76  (0.54-0.77) | 0.64  (0.54-0.81) | 0.60  (0.51-0.80) | 1.00  (0.74-1.00) | 0.75  (0.62-0.85) | 0.20  (0.10-0.26) |
|  | Random Forest | 0.82  (0.40-0.90) | 0.56  (0.47-0.70) | 0.55  (0.46-0.77) | 1.00  (0.53-1.00) | 0.71  (0.52-0.77) | 0.03  (0.27-0.65) |
|  | LASSO | 0.78  (0.49-0.79) | 0.61  (0.47-0.72) | 0.60  (0.46-0.76) | 0.88  (0.57-0.98) | 0.71  (0.53-0.78) | 0.29  (0.24-0.64) |

**Supplementary Table 12: Performance metrics.** Comparison of Random Forest classification performance for the original, SPsimSeq-generated, and LLM-generated gene expression datasets**.**

| **Data Used in Random Forest** | **AUC(%)** | **Accuracy(%)** | **Recall(%)** | **F1(%)** | **Specificity(%)** |
| --- | --- | --- | --- | --- | --- |
| **Real Data** | 0.79  (0.47-1.00) | 0.71  (0.46-1.00) | 0.69  (0.25-1.00) | 0.73  (0.50-1.00) | 0.69  (0.25-1.00) |
| **SPsimSeq** | 0.81  (0.45-1.00) | 0.74  (0.50-0.92) | 0.76  (0.36-1.00) | 0.76  (0.46-0.92) | 0.71  (0.20-1.00) |
| **SPsimSeq-LLM** | 0.94  (0.81-1.00) | 0.88  (0.63-1.00) | 0.89  (0.46-1.00) | 0.89  (0.59-1.00) | 0.87  (0.57-1.00) |

**REFERENCES**

1. Clough, E. and Barrett, T. (2016) The Gene Expression Omnibus database. *Methods in molecular biology (Clifton, N.J.)*, 1418: 93. doi:10.1007/978-1-4939-3578-9_5.
2. Mcinnes, L., Healy, J. and Melville, J. (2020) *UMAP: Uniform Manifold Approximation and Projection for Dimension Reduction*.
3. Page, M.J., McKenzie, J.E., Bossuyt, P.M., et al. (2021) The PRISMA 2020 statement: an updated guideline for reporting systematic reviews. *BMJ*, 372. doi:10.1136/BMJ.N71.
4. Perez-Riverol, Y., Bai, M., Da Veiga Leprevost, F., et al. (2017) Discovering and Linking Public ‘Omics’ Datasets using the Omics Discovery Index. *Nature biotechnology*, 35 (5): 406. doi:10.1038/NBT.3790.
